# Theoretical analysis of lipase-enzymatic DKR model for racemic (R)- and (S)- ibuprofen ester in a hollow cylindrical membrane bioreactor

**DOI:** 10.1038/s41598-025-89583-z

**Published:** 2025-02-15

**Authors:** L. Niyaz Ahmed, Praveen Thomas

**Affiliations:** Department of Mathematics, School of Advanced Sciences, Vellore Institute of Technology, Vellore, 632014 Tamilnadu India

**Keywords:** Racemic ibuprofen ester, Homotopy perturbation method, Mean integrated effectiveness factor, Immobilized enzyme, Convective hydrolysis, Dynamic kinetic resolution, Hollow fiber membrane reactor, Mathematics and computing, Applied mathematics, Biochemistry, Biocatalysis, Enzymes

## Abstract

This study examines the semi-analytical expression of a hollow cylindrical fiber membrane bioreactor catalyzed by an immobilized enzyme (lipase), focusing on the dynamic kinetic resolution (DKR) of racemic (R)- and (S)-ibuprofen ester through the homotopy perturbation method (HPM). This method effectively solves the coupled non-linear system of second-order ordinary differential equations (ODEs), incorporating the mass transfer associated with the DKR reaction rate. The DKR rate equations are computed utilizing both enantiomers, which represent the enzymatic hydrolysis of the substrate within the barrier’s support matrix, where the racemization term is negligible. Additionally, the corresponding racemization of the unreacted substrate outside the membrane is considered, where the enzymatic hydrolysis term is negligible. The dimensionless steady-state solution for the dynamics of the mean integrated effectiveness factor (MIEF) related to convective hydrolysis-racemization phenomena quantifies the local effectiveness factor, which impacts the Thiele modulus and the bulk initial concentrations of a substance within the membrane layer. The Bodenstein number is a critical parameter that optimizes reaction conditions, enhances mixing efficiency, and mitigates mass transfer limitations. The normalized values from the sensitivity analysis are analyzed to identify the key parameters of the present system. The conversion efficiency and limitations of the proposed model utilizing HPM are presented. Our proposed solution compares the semi-analytical expression with the simulated numerical results of BVP-4c using the built-in solver in MATLAB R2023b software, demonstrating good agreement across all values of the reaction parameters. The agreement between semi-analytical and numerical computations demonstrates the validity of these approximation methods. The findings provide significant insights for enhancing membrane reactor design and improving the efficiency of industrial processes for racemic ibuprofen ester production.

**Supplementary Information:**

The online version contains supplementary material available at 10.1038/s41598-025-89583-z.

## Introduction

The (S)-ibuprofen acid exhibited greater than 160 times more reactivity in its analgesic action compared to the (R)-ibuprofen acid. There is rising demand for cost-effective and highly efficient methods to facilitate the industrial manufacturing of pure enantiomers, with a particular emphasis on the synthesis of chiral medicines. The majority of current medications consist only of a single, pure enantiomer. The demand for these optically pure chemicals rises because they have a more specific therapeutic impact compared to racemic combinations. Several conventional approaches generate a combination of both enantiomers, either in racemic form (1:1 molar ratio) or at distinct molar ratios. A prochiral intermediate, precursor, or naturally occurring racemate can establish chiral resolutions^1^. The study examined by L. Goirno et al.^2^. the effectiveness of an immobilized lipase biphasic hollow fiber membrane reactor in the hydrolysis of triglycerides. The following phase was to put the collected data into a system that could produce and separate enantiomeric acids from esters that were in a racemic mixture. The immobilized *Candida cylindracea* lipase (EC 3.1.1.3) using asymmetric hollow fibers of a sponge through cross-flow filtration. Recirculation of both the organic substrate solution and the liquid phase, with the first phase passing through the membranes’ shell side and the second phase passing through the lumen side. The lipase only transformed the S-ester when the substrate consisted of the racemic mixture. The lipase was able to acquire the acid (S)−1, which was soluble in liquid and could be eliminated in the same manner as its preparation, it altered the equilibrium in favor of the product.

A hollow fiber enzymatic membrane reactor employing ultrafiltration was implemented for analyzing the kinetics of the lipase-catalyzed kinetic resolution of racemic ibuprofen ester. *Candida rugosa*lipase was used in the hydrolysis process. It was used both in its free form in a batch system and in its immobilized form in an enzymatic membrane reactor (EMR). The immobilized lipase on the spongy layer has a half-life of 105 h at a reaction temperature of 40 °C and 62 h at 45 °C. The result for lipase immobilized on the inner lumen was 94 h, whereas the value for free lipase in a batch system was 45 h at a temperature of 40 °C. The presence of a substantial quantity of substrate was seen as hindering the reaction as an uncompetitive inhibitor. The by-product 2-ethoxyethanol, when present in excess, behaves as a non-competitive inhibitor in the process. A theoretical study was carried out to examine the process of separating racemates in a batch reactor using an immobilized enzyme. A first-order solution that encompasses both internal and external mass transfers is effectively performed. Consequently, a thorough analysis is needed to measure the impact of both intraparticle and external mass-transfer resistances on the enzyme’s enantioselectivity. Additionally, the implementation of an accurate solution to forecast the progression of substrate concentration, conversion, product purity, and yield over time is noted. In order to forecast the quality and quantity of the final product, the development of a basic estimation that considers both the immobilized enzyme’s concurrent response with external factors and the movement of mass inside the enzyme particles was performed^3–6^.

The hollow-fiber membrane bioreactor was used to carry out the aqueous/organic two-phase DKR process, with the aim of facilitating the preparative scale operation. The procedure consisted of using lipase as a catalyst to selectively form esters in ethylene dichloride while simultaneously using mandelate racemase to convert the unreacted isomer of mandelic acid into its racemic form in an aqueous buffer. The designated regions of this responsive system include the spongy layers, thick membrane, and fiber lumen^7–10^. The chemical reaction should only occur in the skin and sponge, which are the last two regions where the biocatalyst is present and distributed in specific ratios. Through the analysis of two representative parameters, such as the effectiveness factor and the performance index, we were able to evaluate the significant transport mechanisms of the substrate consumption rates and the overall performance of the reactor. This evaluation was made possible by expressing the operating conditions by employing a set of characteristic dimensionless groups. The substrate solution passed through the lumen of the reactor, while the enzyme was physically enclosed inside the reactor’s shell. The reaction products and substrates permeated the fiber wall by diffusion^10–14^. To establish a two-enzyme DKR system, one might employ enzyme-catalyzed in situ racemization in addition to the typical enzymatic resolution phase. This method employs enzymes that carry out in situ racemization, potentially facilitating the combination of milder reaction conditions with those necessary for the enzyme to catalyze kinetic resolution. In biocatalytic dynamic kinetic resolution (DKR), one of the hardest things to do is to find conditions that racemize the substrate while keeping the enzyme active during the kinetic resolution process^15^. The dynamic kinetic resolution (DKR) of racemic ibuprofen ester, which is essential in the synthesis of this prominent pharmaceutical compound, has garnered attention for its capacity to produce valuable enantiomers effectively. Despite the progress made, various challenges remain, primarily arising from the complexity of reaction kinetics and mass transfer limitations within reactor designs, such as hollow fiber membrane reactors. Recent advancements in mathematical modeling provide valuable frameworks to analyze and optimize such biological systems. Nonetheless, traditional approaches often fall short in accurately capturing the nonlinear dynamics that characterize these systems. In this present study, a comprehensive investigation into the DKR of racemic ibuprofen ester using a robust analytical framework based on the homotopy perturbation method (HPM).

To solve the non-linear systems of ordinary differential equations (ODEs) that describe the diffusion phenomena associated with Michaelis-Menten (MM) kinetics, there are several semi-analytical approaches such as Akbari-Ganji’s method (AGM), modified Adomian decomposition method (MADM), variational iteration method (VIM), Taylor series method (TSM), and hyperbolic function method (HFM) that have been widely used^16–21^. L. Niyaz Ahmed and Praveen T discussed the AGM technique for solving the reaction-diffusion (RD) model (ODEs)^22^. This approach relies on selecting appropriate base functions as an initial guess, which greatly influences the accuracy and convergence of the solution. However, predicting the optimal base functions can be challenging in some cases. Praveen T et al.^23^discussed the modified Adomian decomposition method (MADM), which is a technique where a nonlinear equation is decomposed into a series of linear sub-equations. However, in strongly nonlinear problems, the convergence of the solution process may slow down, and the accuracy of the solution is dependent on the initial guess. The variational iteration method (VIM) exhibits convergence behavior that varies depending on the initial approximation chosen for the problem, as studied by Wazwaz and Abdul-Majid^24^. L. Rajendran et al. explored that the Taylor series method (TSM) may not always be suitable for solving nonlinear equations, whereas the hyperbolic function method (HFM) is not universally applicable due to the requirement of transforming nonlinear ordinary differential equations (ODEs) into a linear type with hyperbolic coefficients^25,26^. Unlike other semi-analytical approaches, the homotopy perturbation method (HPM) is a powerful technique to examine the behavior of concentration profiles for various values of the kinetic parameters. The HPM approach is quite a reliable and efficient method that treats the embedding parameter *p* as a small artificial parameter. It is possible to determine an asymptotic, simple, and closed-form solution with a minimal number of iterations. The HPM technique provides solutions in the form of a convergent series.

C. M. Mohana and B. Rushi Kumar^27,28^ discussed the coupled nonlinear fluid flow problems using built-in MATLAB R2023b software using BVP-4c along with the shooting technique (an implicit Runge-Kutta method), which is a reliable technique where collocation polynomial ensures ${C^1}$continuity of the solution and mesh selectivity in order to obtain the solution’s accuracy and requires good initial guesses for successful computations other than any numerical techniques^29–31^. In our present investigation, we adapt the HPM semi-analytical approach^21,33–35^, which suits perfectly to the current study as compared to the other asymptotic approximate techniques, and BVP-4c utilization, apart from any other numerical solver.

Examining the chemical reaction that is inhibited by the presence of both the product and the substrate, the present work aims to use the optimal operating parameters that result in the maximum productivity of the system by analyzing the behavior of the transport processes occurring in a hollow cylindrical fiber membrane reactor.

The objective of this investigation is carried out as follows:

- Under the steady-state regime, with the aid of the HPM approach, the coupled nonlinear differential equation system is analytically computed for the dimensionless concentration of racemic (R)- and (S)-ibuprofen ester, respectively.
- To validate the asymptotic semi-analytical homotopy perturbation approach by comparing the simulated numerical results using BVP-4c along with the shooting technique for the concentration profiles of (R)- and (S)-ibuprofen esters under equilibrium conditions.
- The dynamics of MIEF using the HPM technique are carried out to analyze the impact of the Thiele modulus and the initial bulk concentration of substrate in the system.
- The limitations of the valid and invalid zones have been portrayed for MIEF, and the performance of normalized sensitivity analysis undertaking the various important operating parameters in order to check which parameter shows the highest influence, respectively, on our proposed model.

## Formulation of the mathematical modeling

**Fig. 1:**
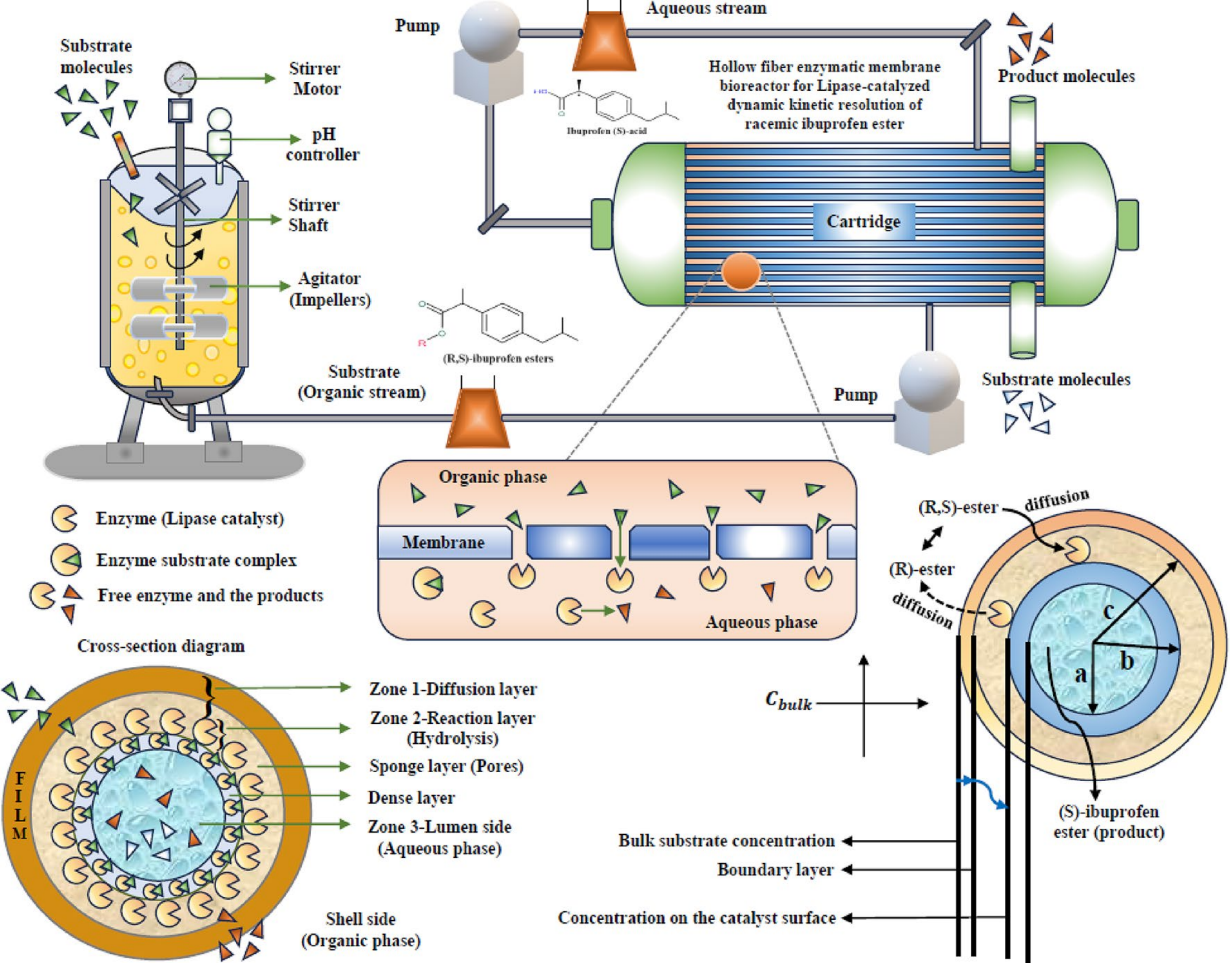
Graphical representation of lipase-catalyzed of DKR racemic ibuprofen ester.

The mathematical modelling of the hollow fiber membrane reactor involving the lipase-catalyzed immobilized enzyme of DKR system^7,13^ is depicted in Fig. 1. This model entails two different phenomena inside the reactor such, as lumen (aqueous) and shell (organic) mediums respectively. The membrane’s hydrophilic nature restricts the organic phase from interacting with the aqueous phase. As a result, both the excess racemic ester and the unreacted substrate ester ((R)-ibuprofen ester) ended up on the shell side of the membrane matrix after the hydrolysis process. At the same time, the (R)-ibuprofen ester goes through racemization on the shell side while tri-octylamine, which works as a base catalyst. Only the product ((S)-ibuprofen acid), which has a high solubility in the aqueous phase, is able to pass through the membrane and enter the lumen side. Consequently, the product and substrate may be readily separated. The hollow fiber membrane facilitates the DKR process, which involves diffusion, hydrolysis, and racemization. The following assumptions were used during the model development: (i) The enzyme directly interacts with the organic substrate; (ii) The enzyme is evenly distributed on the membrane surface; (iii) The reaction maintains a constant temperature throughout the hollow fiber, (iv) The effective diffusivity of the substrate and product remains constant; and (v) The accumulation of the by-product, alcohol, dropped was presented by Subash Bhatia et al.^12,13^, which is portrayed in the following mechanism.


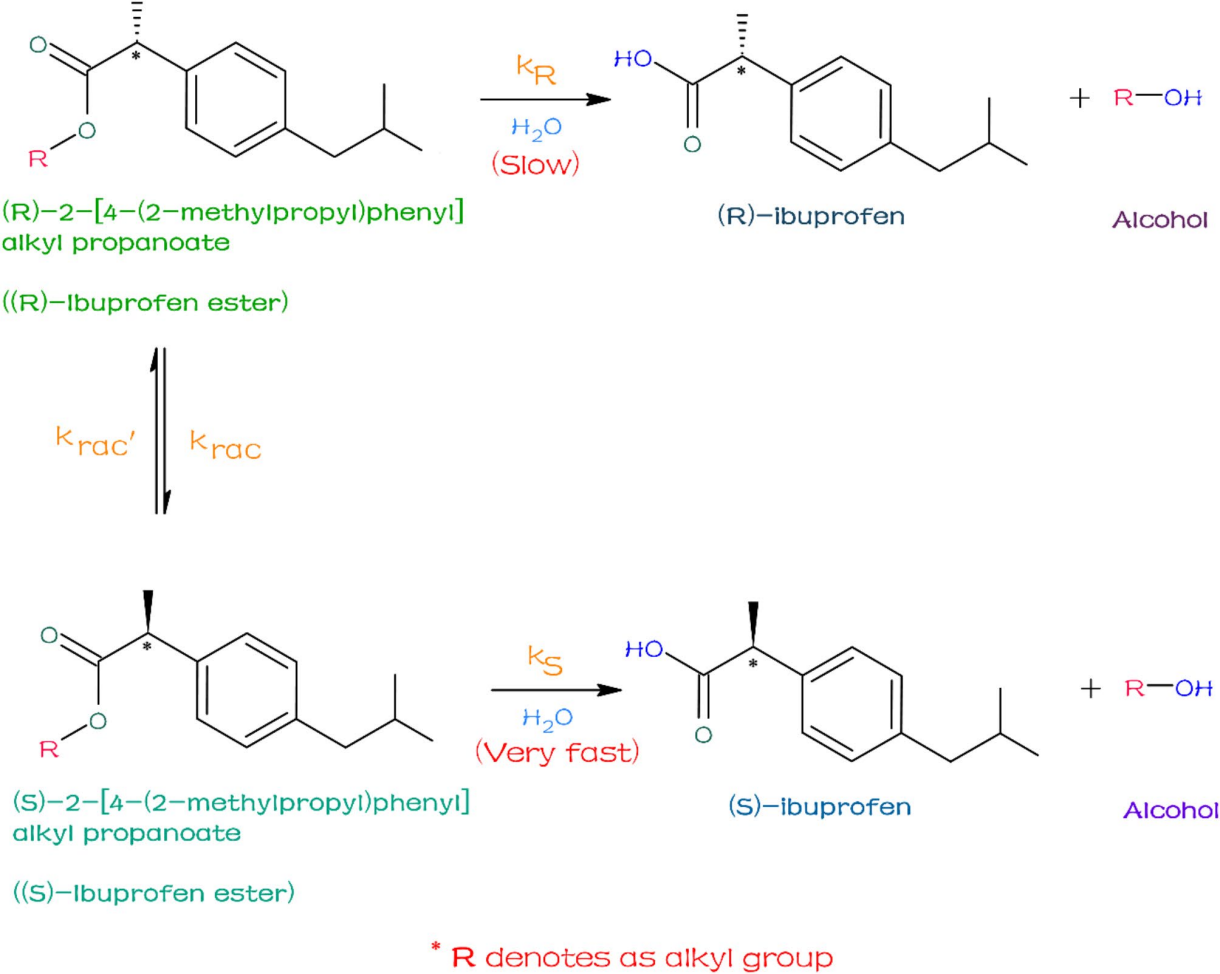


The inhibitor binds both the free enzyme *E* and the enzyme-substrate complex *ES*, the kinetic constant terms for the types of inhibitors are non-competitive inhibitor (${K_{nI1}},{K_{nI2}}$), and uncompetitive substrate inhibitor (${K_{uS1}},{K_{uS2}}$), respectively. In order to represent the distribution of the total enzyme *[E]*_*T*_, the mass balance equation may be expressed as follows:

1 $$[E]_T= [E] + [ES] + [ES^*] + [EI] + [ESS^*] + [ESI],\\ v=\frac{d[P']}{dt}=k_{\text{cat}}[ES]$$

where $v$ signifies the formed product initial rates. The modified DKR rate equations are then followed by^13^:

2 $$v=\frac{{{v_{\hbox{max} }}\,[S]}}{{\left( {{K_m}+[S]} \right)\left( {1+\frac{{[I]}}{{{K_{nI1}}}}+\frac{{[S*]}}{{{K_{uS1}}}}} \right)}}.$$

The reaction phenomena take place inside the membrane porous support, and the rate of reaction for both (R)- and (S)- enantiomers are given as follows:

3 $$\left. \begin{gathered} {v_A}= - \frac{{d{s_A}}}{{dt}}=\frac{{{v_{\hbox{max} }}\,{s_A}}}{{\left( {{K_{mA}}+{s_A}} \right)\left( {1+\frac{{{s_I}}}{{{K_{nI1}}}}+\frac{{{s_B}}}{{{K_{uS1}}}}} \right)}} \hfill \\ {v_B}= - \frac{{d{s_B}}}{{dt}}=\frac{{{v_{\hbox{max} }}\,{s_B}}}{{\left( {{K_{mB}}+{s_B}} \right)\left( {1+\frac{{{s_I}}}{{{K_{nI1}}}}+\frac{{{s_A}}}{{K_{{uS1}}^{\prime }}}} \right)}} \hfill \\ \end{gathered} \right\},$$

where ${v_A},{s_A},{v_{\hbox{max} }},{K_{mA}},K_{{uS1}}^{\prime }$, are reaction rate constants, substrate concentration, maximum reaction rate constant, Michaelis-Menten constant, uncompetitive substrate inhibition constant of (S)-enantiomers and ${v_B},{s_B},{v_{\hbox{max} }},{K_{mB}},{K_{uS1}}$, are reaction rate constants, substrate concentration, maximum reaction rate constant, Michaelis-Menten constant, uncompetitive substrate inhibition constant of (R)-enantiomers and ${s_I}$denotes the non-competitive alcohol inhibitor concentration.

The steady-state racemization of unreacted substrate components at the bulk phase and the rate of change of (R)- and (S)-enantiomers concentrations are stated as follows:

4 $$\left. \begin{gathered} {v_A}= - \frac{{d{s_A}}}{{dt}}=\frac{{{V_{eq}}}}{2}\left[ {\frac{{{s_A} - {s_B}}}{{{s_A}+{s_B}}}} \right] \hfill \\ {v_B}= - \frac{{d{s_B}}}{{dt}}=\frac{{{V_{eq}}}}{2}\left[ {\frac{{{s_B} - \,{s_A}}}{{{s_A}+{s_B}}}} \right] \hfill \\ \end{gathered} \right\},$$

where ${V_{eq}}$ is the dynamic equilibrium rate constant.

The complete DKR model evolved considering the following assumptions: (i) only radial diffusion is taken into account as the axial gradient is considered to be insignificant; (ii) Fick’s law is carried out; (iii) the fluid properties, such as density and viscosity, remain unchanged across the membrane; and (iv) the dead zone is abandoned. The mass transfer equation for the ester of (S)-ibuprofen was formulated by incorporating a differential equation that adheres to the specified boundary conditions^13^:

5 $$u\left( r \right)\frac{{d{s_i}}}{{dr}}+{D_{eff}}\left[ {\frac{{{d^2}{s_i}}}{{d{r^2}}}+\frac{1}{r}\frac{{d{s_i}}}{{dr}}} \right]={\alpha _p}{v_i};\,\,i=A,B,$$

where ${s_i}$ denotes the concentration of racemic substrate ester, ${\alpha _p}$ is a measure of porosity membrane, ${D_{eff}}$ is the effective diffusivity, the enzymatic reaction rate is denoted as ${v_i}$, $u\left( r \right)\,\frac{{d{s_i}}}{{dr}}$; is a convective mass transfer and the radial flow velocity is denoted as $u\left( r \right)$, and is expressed as $u\left( r \right)\,=\frac{F}{{2\pi rLN}}$; notice that here, $F,L,N,r$denotes the substrate stream volumetric flow rate, length of effective fiber, number of fibers and fiber radius respectively.

The boundary conditions along the boundary line (external fiber radius/surface concentration radius) $(r=c)$ and the centerline symmetry ($r=b$) are displayed as^13^:

6 $$\left. \begin{gathered} \,\frac{{d{s_i}}}{{dr}}=0\,\,\,\,\,\,\,\,\,\,\,\,\,\,\,\,\,\,\,\,\,\,\,\,\,\,\,\,\,\,\,\,\,\,\,\,at\,\,\,r=b \hfill \\ {s_{i0}}={s_i}+\frac{{{D_{eff}}}}{{u\left( r \right)}}\left( {\frac{{d{s_i}}}{{dr}}} \right)\,\,\,\,\,\,\,\,\,\,at\,\,\,r=c \hfill \\ \end{gathered} \right\}$$

From the Eq. (3) and Eq. (4), Sie Yon Lau et al. depicts the model equations which were simplified and is converted into the dimensionless form of the Eq. 1^**3**^.

7a $${v_A}= - \frac{{d{s_A}}}{{d\tau }}=\frac{{{\Psi _A}\,{s_A}}}{{\left( {{\Theta _A}+{s_A}} \right)\left( {1+\phi {\xi _{IP}}+{\xi _{IS}}{s_B}} \right)}}+\frac{{{V_{eq}}}}{2}\left[ {\frac{{{s_A} - {s_B}}}{{{s_A}+{s_B}}}} \right],$$

7b $${v_B}= - \frac{{d{s_B}}}{{d\tau }}=\frac{{{\Psi _B}\,{s_A}}}{{\left( {{\Theta _B}+{s_B}} \right)\left( {1+\phi {\xi _{IP}}+\xi {_{IS}}{s_A}} \right)}}+\frac{{{V_{eq}}}}{2}\left[ {\frac{{{s_B} - \,{s_A}}}{{{s_A}+{s_B}}}} \right]$$

where, the dimensionless terms of (S)-Enantiomer and (R)-Enantiomer are as follows: $\forall \,\,i=A,B,$ ${\Psi _i}=\frac{{{\Theta _i}{t_0}{v_{\hbox{max} }}}}{{{K_{mi}}}}$is enzymatic DKR constant, ${\Theta _i}=\frac{{{K_{mi}}}}{{{s_{i0}}}}$is Michaelis-Menten constant, ${\xi _{IS}}=\frac{{{s_{B0}}}}{{{K_{uS1}}}}$, $\xi^{'}_{IS}=\frac{s_{A0}}{K^{'}_{uS1}}$are substrate inhibition constant, ${\xi _{IP}}=\frac{{{s_{by}}}}{{{K_{nI1}}}}$is by-product inhibition constant, and $\phi =\frac{{{s_{I0}}}}{{{s_{A0}}}}$is product inhibition fraction^13^.

Since, the rate of hydrolysis of (S)-ibuprofen ester is very much stronger than (R)-ibuprofen ester and hence, ${v_A}$term in the Eq. (7a) is eliminated (i.e., the term ${V_{eq}} \to 0$). Similarly, racemization rate overshoots the hydrolysis rate and therefore, ${v_B}$term in the Eq. (7b) is neglected (i.e., the term ${\psi _B} \to 0$). By converting and solving the equations (Eq. (3) – Eq. (6)) into a dimensionless form.

The dimensionless quantities are stated as follows:

8 $$\left. \begin{gathered} {S_1}=\frac{{{s_A}}}{{{s_{A0}}}},\,\,{S_2}=\frac{{{s_B}}}{{{s_{B0}}}},\,\,x=\frac{r}{a},X=\frac{{{s_{i0}} - {s_i}}}{{{s_{i0}}}},\,{S_{ib}}=\frac{{{S_i}}}{{1 - X}};\,i=1,2 \hfill \\ {\Phi ^2}=\frac{{{a^2}\,{\alpha _p}\,{v_{\hbox{max} }}}}{{{D_{eff}}\,{K_{mA}}}},{B_0}=\frac{{r\,u\left( r \right)}}{{{D_{eff}}}},\,\,\Theta \,=\frac{{{K_{mA}}}}{{{s_{A0}}}},\,\,{\xi _1}=\frac{{{s_{B0}}}}{{{K_{uS1}}}},{\xi _2}=\frac{{{s_{A0}}}}{{{K_{nI1}}}}, \hfill \\ \phi =\frac{{{s_I}}}{{{s_{A0}}}},\,\,\gamma =\frac{{{a^2}\,{r_{rac}}}}{{{D_{eff}}\,{s_{T0}}}},\,\,{s_{T0}}=2{s_{B0}},\,\,{V_{eq}}={r_{rac}}={K_{rac}}\,{s_{OH}}, \hfill \\ \end{gathered} \right\}$$

From the above Eq. (8), the dimensionless groups are: ${S_1}$ is the dimensionless concentration of (S)-ibuprofen ester, ${S_2}$ is the dimensionless concentration of (R)-ibuprofen ester, ${S_{ib}}$ is the dimensionless radial distance, $X$ is the conversion of both (S) and (R)-ibuprofen esters, ${S_{ib}}$ is the dimensionless initial bulk concentrations $i=1,2,$ ${\Phi ^2}$ is Thiele modulus for the (S)-ibuprofen ester, ${B_0}$ is the Bodenstein number, $\Theta$ is the Michaelis-Menten constant for (S)-ibuprofen ester, $\,{\xi _1}$ is the dimensionless substrate inhibition constant for (R)-ibuprofen ester, ${\xi _2}$ denotes the dimensionless by-product inhibition constant for alcohol, $\phi$ denotes the molar fraction of by-product and substrate inhibition constant, $\gamma$ is the dimensionless racemization constant, and ${s_{T0}}$is the initial concentration of racemic ibuprofen ester. Moreover, the dimensionless quantity Bodenstein number is expressed as^13^

9 $${B_0}=\frac{{r\,u\left( r \right)}}{{{D_{eff}}}}=\frac{{{\text{total momentum transfer}}}}{{{\text{molecular mass transfer}}}}$$

Therefore, the newly transformed dimensionless governing second-order ordinary differential equations, which are coupled and strongly nonlinear associated with the boundary conditions by considering the mass transfer of both substrate (S)-ester from bulk to membrane matrix and the unreacted substrate (R)-ester from the membrane matrix to the bulk concentration respectively as follows:

The governing rate equation for the dimensionless concentration of (S)-ibuprofen ester at both shell and membrane matrix phase involving the reaction process of hydrolysis are

10 $$\frac{{{d^2}{S_1}}}{{d{x^2}}}+\frac{1}{x}\left( {1+{B_0}} \right)\frac{{d{S_1}}}{{dx}}=\frac{{{\Phi ^2}{S_1}}}{{\left( {1+\frac{{{S_1}}}{\Theta }} \right)\left( {1+\phi {\xi _2}+{\xi _1}{S_2}} \right)}}.$$

The governing rate equation for the dimensionless concentration of (R)-ibuprofen ester covering both diffusion and racemization phase are

11 $$\frac{{{d^2}{S_2}}}{{d{x^2}}}+\frac{1}{x}\left( {1+{B_0}} \right)\frac{{d{S_2}}}{{dx}}=\gamma \left( {\frac{{{S_1} - {S_2}}}{{{S_1}+{S_2}}}} \right)$$

Associated boundary conditions are:

12 $$\frac{{d{S_1}\left( x \right)}}{{dx}}=\frac{{d{S_2}\left( x \right)}}{{dx}}=0\,\,\,\,\,\,\,{\text{at}}\,\,x=1$$

Since, from the external fiber radius Eq. (6), after converting into dimensionless state, the boundary condition at $x=2$ becomes,

$\frac{{d{S_i}\left( x \right)}}{{dx}}=\frac{{{B_0}}}{x}\,\left( {1 - {S_i}\left( x \right)} \right),\,\,\forall \,\,i=1,2$, and from this we get,

$\Rightarrow {S_i}\left( x \right)=1 - \frac{1}{{{x^{{B_0}}}}}\,,\,\,\forall \,\,i=1,2$, and hence, for some fixed values of ${B_0}$(Bodenstein number)^5^, the required boundary condition (refer **Appendix D**) is as follows.

13 $${S_1}\left( x \right)\approx{S_2}\left( x \right)\approx1\,\,\,\,\,\,\,\,\,{\text{at }}\,x=2$$

## Materials and methods

### Advantages of homotopy perturbation method (HPM)

As discussed earlier in the introduction section, we contrasted the various analytical and numerical approaches for solving nonlinear differential equations, although solving nonlinear differential equations is extremely hard. This article focuses on Homotopy perturbation technique^16–21^, a new asymptotic series approximation approach (also known as the semi-analytical method). This simple and closed-form approach explains how to tackle the non-linear problems, including non-vibrational, vibrational, reaction-diffusion, and integro-differential equations. This method is more computationally efficient and can provide a high degree of accuracy with just two to three iterations. The method often exhibits faster convergence with a reduced number of iterations when compared to other semi-analytical methods. The series solution generally exhibits rapid convergence towards the exact solution^33–36^.

The Homotopy Perturbation Method (HPM) was selected for its distinct advantages over other approaches^22–26^, particularly in terms of computational efficiency and accuracy. Compared to the Variational Iteration Method, which heavily depends on initial approximations and can become cumbersome in complex cases, HPM is less reliant on such initial guesses, making it more adaptable to a wider range of problems. Unlike the Taylor Series Method, which struggles with handling nonlinearities in differential equations, HPM excels in addressing nonlinear systems, including the coupled non-linear system of second-order differential equations that characterize the membrane reactor model. Furthermore, HPM demonstrates greater simplicity and speed than the Adomian Decomposition Method, which requires complex recursive terms that may increase the computational load. The Hyperbolic Function Method, while effective for specific functional forms, lacks the general applicability and rapid convergence that HPM provides, especially for complex reaction-diffusion systems. Similarly, Akbari-Ganji’s Method^36^, though useful for obtaining closed-form solutions, can be less efficient and flexible in handling intricate nonlinearities. Thus, HPM’s rapid convergence, minimal iterations, and ability to yield straightforward, closed-form solutions establish it as an ideal method for this study.

### A symptotic approximate expression for the dimensionless concentration of (S)-ibuprofen ester and (R)-ibuprofen ester by employing the semi-analytical HPM technique under the steady-state regime

In this study, we opted for a semi-analytical technique, the homotopy perturbation method (see **Appendix A**), to solve the system of coupled non-linear second-order BVP. The series form of analytical expression for the dimensionless concentration of (S)- and (R)-ibuprofen ester (see **Appendix B**) of the substrates ${S_1}(x),\,\,{S_2}(x)$respectively, given as,

14 $$\begin{gathered} {S_1}\left( x \right)=1+\frac{{{\Phi ^2}\,\Theta \left( {{x^2}{B_0} - 4{B_0} - {2^{1 - {B_0}}}+2{x^{ - {B_0}}}} \right)\,}}{{2{B_0}\left( {2+{B_0}} \right)\left( {1+\Theta } \right)\left( {1+\phi {\xi _2}+{\xi _1}} \right)}} \\ +\frac{{{\Phi ^4}\,{\Theta ^3}\left( \begin{gathered} \left( { - 4B_{0}^{3} - 24B_{0}^{2} - 32{B_0}} \right){x^{2 - {\beta _0}}}+\left( \begin{gathered} \left( { - 8B_{0}^{2} - 16{B_0}+64} \right){x^{ - {B_0}}} \hfill \\ - 4{x^2}B_{0}^{3} - 8{x^2}B_{0}^{2}+32{x^2}{B_0}+40B_{0}^{3}+144B_{0}^{2} - 96{B_0}+64 \hfill \\ \end{gathered} \right){2^{ - {B_0}}} \hfill \\ +\left( { - 2+{B_0}} \right)\left( {\left( { - 8B_{0}^{2} - 32{B_0}+32} \right){x^{ - {B_0}}}+\left( {8{B_0}+32} \right){4^{ - {B_0}}}+\left( {\left( {{x^2} - 4} \right){B_0}+2{x^2} - 24} \right)\left( {{x^2} - 4} \right)B_{0}^{2}} \right) \hfill \\ \end{gathered} \right)}}{{\left( {8B_{0}^{2}\left( {4+{{\text{B}}_0}} \right)\left( { - 2+{{\text{B}}_0}} \right){{\left( {2+{{\text{B}}_0}} \right)}^2}{{\left( {1+\Theta } \right)}^3}{{\left( {1+\phi {\xi _2}+{\xi _1}} \right)}^2}} \right)}} \\ \end{gathered}$$

15 $${S_2}\left( x \right)=1+\frac{{\gamma \,{\Phi ^2}\,\Theta \left( \begin{gathered} \left( { - 3B_{0}^{2} - 4{{\text{B}}_0} - \frac{1}{2}B_{0}^{3}} \right){x^{2 - {{\text{B}}_0}}}+\left( \begin{gathered} \left( { - B_{0}^{2} - 2{{\text{B}}_0}+8} \right){x^{ - {{\text{B}}_0}}} \hfill \\ - \frac{{{x^2}B_{0}^{3}}}{2} - {x^2}B_{0}^{2}+4{x^2}{{\text{B}}_0}+5B_{0}^{3}+18B_{0}^{2} - 12{{\text{B}}_0}+8 \hfill \\ \end{gathered} \right){2^{ - {{\text{B}}_0}}} \hfill \\ +\left( { - 2+{{\text{B}}_0}} \right)\left( {\left( { - B_{0}^{2} - 4{{\text{B}}_0}+4} \right){x^{ - {{\text{B}}_0}}}+\left( {{{\text{B}}_0}+4} \right){4^{ - {{\text{B}}_0}}}+\frac{{\left( {\left( {{x^2} - 4} \right){{\text{B}}_0}+2{x^2} - 24} \right)\left( {{x^2} - 4} \right)B_{0}^{2}}}{8}} \right) \hfill \\ \end{gathered} \right)}}{{\left( {2B_{0}^{2}\left( {4+{{\text{B}}_0}} \right)\left( { - 2+{{\text{B}}_0}} \right){{\left( {2+{{\text{B}}_0}} \right)}^2}\left( {1+\Theta } \right)\left( {1+\phi {\xi _2}+{\xi _1}} \right)} \right)}}$$

#### The solution expression using the semi-analytical HPM technique for the dimensionless concentration of a substance with the degree of conversion

Let us consider the conversion of overall hydrolysis, which was discussed by Subhash Bhatia et al.^5^. The conversion of dimensionless concentration of (S)-ibuprofen ester ${S_1}$ with respect to the dimensionless initial bulk concentration of (S)-ester ${S_{1b}}$ is expressed as

16 $$X=\frac{{{S_{1b}} - {S_1}}}{{{S_{1b}}}}$$

The concentration of (S)-ester in reference to the conversion degree term is as follows

17 $$\begin{gathered} {S_1}\left( x \right)=1 - \frac{{{\Phi ^2}\,\Theta \left( { - 0.5\,{x^2}{{\text{B}}_0}+2{{\text{B}}_0}+{2^{ - {{\text{B}}_0}}} - {x^{ - {{\text{B}}_0}}}} \right)\,}}{{{{\text{B}}_0}\left( {2+{{\text{B}}_0}} \right)\left( {\left( {X - 1} \right){S_{1b}} - \Theta } \right)\left( { - 1 - \phi {\xi _2}+\left( {X - 1} \right){S_{2b}}{\xi _1}} \right)}} \\ - \frac{{{\Phi ^4}\,{\Theta ^2}\left( \begin{gathered} \left( {0.5B_{0}^{3}+3B_{0}^{2}+4{{\text{B}}_0}} \right){x^{2 - {{\text{B}}_0}}}+\left( \begin{gathered} \left( {B_{0}^{2}+2{{\text{B}}_0} - 8} \right){x^{ - {{\text{B}}_0}}} \hfill \\ +0.5{x^2}B_{0}^{3}+{x^2}B_{0}^{2} - 4{x^2}{{\text{B}}_0} - 5B_{0}^{3} - 18B_{0}^{2}+12{{\text{B}}_0} - 8 \hfill \\ \end{gathered} \right){2^{ - {{\text{B}}_0}}} \hfill \\ - \left( { - 2+{{\text{B}}_0}} \right)\left( {\left( { - B_{0}^{2} - 4{{\text{B}}_0}+4} \right){x^{ - {{\text{B}}_0}}}+\left( {{{\text{B}}_0}+4} \right){4^{ - {{\text{B}}_0}}}+0.125\left( {\left( {{x^2} - 4} \right){{\text{B}}_0}+2{x^2} - 24} \right)\left( {{x^2} - 4} \right)B_{0}^{2}} \right) \hfill \\ \end{gathered} \right)}}{{\left( {B_{0}^{2}\left( {4+{{\text{B}}_0}} \right)\left( { - 2+{{\text{B}}_0}} \right){{\left( {2+{{\text{B}}_0}} \right)}^2}{{\left( {\left( {X - 1} \right){S_{1b}} - \Theta } \right)}^2}{{\left( { - 1 - \phi {\xi _2}+\left( {X - 1} \right){S_{2b}}{\xi _1}} \right)}^2}} \right)}} \\ \end{gathered}$$

And the concentration of (R)-ester in reference to the conversion degree term is as follows.

18 $$\begin{gathered} {S_2}\left( x \right)=1+\frac{{\left( {\,\frac{{\gamma {x^2}\left( {1+\left( {X - 1} \right){S_{1b}}} \right)}}{2}+\frac{{\gamma \left( {1+{S_{1b}}\left( {X - 1} \right)} \right)\left( {\left( {2+{B_0}} \right)\left( {X - 1} \right)\left( {{S_{2b}}+{S_{1b}}} \right){x^{ - {B_0}}} - \left( {{2^{ - {B_0}}}+2{B_0}} \right)} \right)}}{{{B_0}\left( {{S_{2b}}X{B_0}+{S_{1b}}X{B_0}+2{S_{2b}}X+2{S_{1b}}X - {S_{2b}}{B_0} - {S_{1b}}{B_0} - 2{S_{2b}} - 2{S_{1b}}} \right)}}} \right)}}{{\left( {2+{{\text{B}}_0}} \right)\left( {X - 1} \right)\left( {{S_{1b}}+{S_{2b}}} \right)}} \\ - \frac{{{\gamma ^2}\left( {1+{S_{1b}}\left( {X - 1} \right)} \right)\left( \begin{gathered} \left( {B_{0}^{3}+6B_{0}^{2}+8{{\text{B}}_0}} \right){x^{2 - {{\text{B}}_0}}}+\left( \begin{gathered} \left( {2B_{0}^{2}+4{{\text{B}}_0} - 16} \right){x^{ - {{\text{B}}_0}}} \hfill \\ +{x^2}B_{0}^{3}+2{x^2}B_{0}^{2} - 8{x^2}{{\text{B}}_0} - 10B_{0}^{3} - 36B_{0}^{2}+24{{\text{B}}_0} - 16 \hfill \\ \end{gathered} \right){2^{ - {{\text{B}}_0}}} \hfill \\ - \left( { - 2+{{\text{B}}_0}} \right)\left( \begin{gathered} \left( { - 2B_{0}^{2} - 8{{\text{B}}_0}+8} \right){x^{ - {{\text{B}}_0}}}+\left( {{\text{2}}{{\text{B}}_0}+8} \right){4^{ - {{\text{B}}_0}}} \hfill \\ +0.25\left( {\left( {{x^2} - 4} \right){{\text{B}}_0}+2{x^2} - 24} \right)\left( {{x^2} - 4} \right)B_{0}^{2} \hfill \\ \end{gathered} \right) \hfill \\ \end{gathered} \right)}}{{\left( {2B_{0}^{2}\left( {4+{{\text{B}}_0}} \right)\left( { - 2+{{\text{B}}_0}} \right){{\left( {2+{{\text{B}}_0}} \right)}^2}{{\left( {\left( {X - 1} \right)\left( {{S_{1b}}+{S_{2b}}} \right)} \right)}^2}} \right)}} \\ \end{gathered}$$

## The global integrated effectiveness factor

In recent years, the applications in the fields of biology and engineering often involve the study of steady-state, nonlinear diffusion equations. These equations are used to describe the reactions that occur in enzyme solutions that are confined or restricted in a specific manner. The Thiele modulus can be employed to effectively show the impact of external mass transfer resistances and substrate concentrations on the overall effectiveness factor of an enzyme catalyst particle^12^.

For a cylindrical model geometry, the definition for the global mean integrated effectiveness factor is stated mathematically as^7^,

19 $${\eta _m}=\frac{{2\,\int\limits_{{{r_i}}}^{{{r_0}}} {x\,\eta \left( x \right)\,dx} }}{{r_{0}^{2} - r_{i}^{2}}}$$

where, ${r_0}$ is an outer radius of the hollow cylinder and ${r_i}$ is the inner radius of the hollow cylinder.

The local effectiveness factor, according to the reaction rate equation, is widely defined as:

20 $$\eta =\frac{{{\text{reaction rate inside the biocatalyst particle }}}}{{{\text{reaction rate at catalytic surface conditions (bulk phase)}}}}=\frac{{v(S(r))}}{{v({S_b})}}$$

For the lipase-catalyzed of DKR racemic ibuprofen ester, the local effectiveness factor is derived from the Eq. (20) as,

21 $$\eta \left( x \right)=\frac{{{S_1}\left( x \right)}}{{{{\left. {{S_1}\left( x \right)} \right|}_{\left( {{\text{at the bulk interface}}} \right)}}}}$$

### Mean integrated effectiveness factor $\eta_{m}$ for the overall bioreactor performance as a function of Thiele modulus $\Phi$ using HPM solution

The mean integrated effectiveness factor is derived for the solution of (S)-ester concentration using the Eq. (19) and Eq. (21) are as follows:

22 $${\eta _m}=\frac{{ - \left( {\Theta +{S_{1b}}} \right)\left( {1+{S_{2b}}{\xi _1}+\phi {\xi _2}} \right)\left( \begin{gathered} 3\,\left( {{2^{ - {{\text{B}}_0}}}} \right)\,{{\text{B}}_0}\,{\Theta ^2}\,{\Phi ^2} - 3\left( {1+{\xi _1}+\phi {\xi _2}} \right){\left( {\Theta +1} \right)^2}B_{0}^{3} \hfill \\ +\left( {12{{\left( {\Theta +1} \right)}^2}{\xi _1}+12\phi {\xi _2}{{\left( {\Theta +1} \right)}^2}+\left( {12 - \frac{{9{\Phi ^2}}}{4}} \right){\Theta ^2}+24\Theta +12} \right){B_0} \hfill \\ +\left( {{2^{1 - {B_0}}} - 2} \right)\,{\Theta ^2}\,{\Phi ^2}+\frac{{9\left( {{\Theta ^2}\,{\Phi ^2}B_{0}^{2}} \right)}}{4} \hfill \\ \end{gathered} \right)}}{{\left( {3\,{{\text{B}}_0}\,{S_{1b}}\left( {{{\text{B}}_0}+2} \right)\left( {{{\text{B}}_0} - 2} \right){{\left( {1+{\xi _1}+\phi {\xi _2}} \right)}^2}{{\left( {\Theta +1} \right)}^3}} \right)}}$$

## Results and discussion

In this present study, we consider the most important operating parameters^3,5,13,14^ (see **Table 1**) in order to gauge the potency of the model through the semi-analytical HPM technique for both the concentration profiles and the mean integrated effectiveness factor of the system. We further analyze the limitation/validity zones of our proposed semi-analytical expression in the constraint region locally. Moreover, the normalized sensitive analysis performance of our model is carried out, and we compared the solutions between HPM and BVP-4c, which are found to be in good agreement for the distinct values of the operating parameters Thiele modulus (${\Phi ^2}$), Bodenstein number (${B_0}$), and the racemization constant ($\gamma$).

## A dimensionless concentration profile throughout the porous membrane support of the substrates (S)-ibuprofen and (R)-ibuprofen esters across the membrane matrix (1<x<2) for the effect of Bodenstein number $(\rm{B}_{0})$ under the steady-state assumptions

**Fig. 2:**
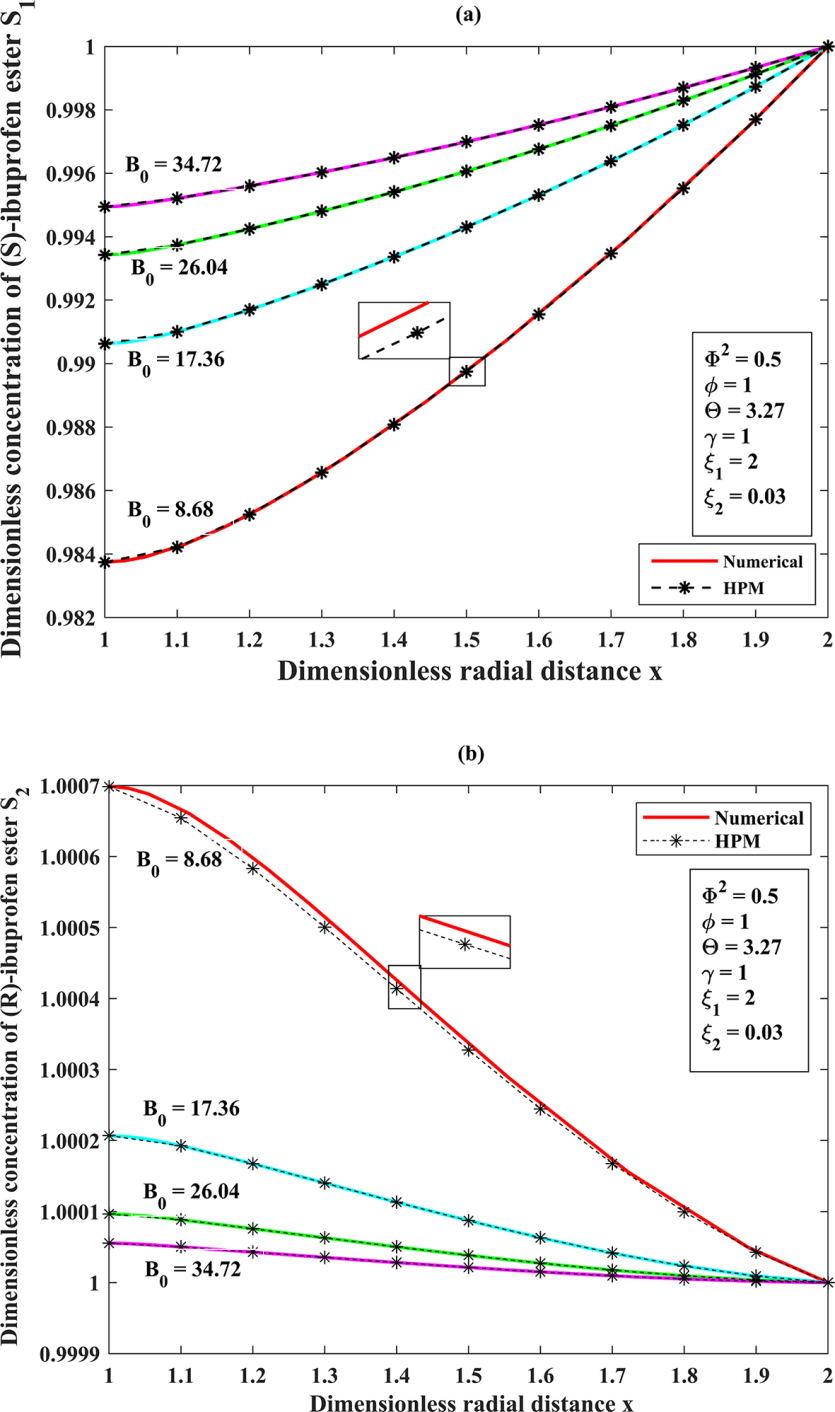
(**a**) Comparison of semi-analytical (HPM) dimensionless concentration of (S)-ibuprofen ester ${S_1}(x)$ versus dimensionless radial distance $S_2(x)$ with numerical simulation results (bvp-4c) for the various Bodenstein number ${B_0}$ using the Eqs. (10), and (12). (**b**) Comparison of semi-analytical (HPM) dimensionless concentration of (R)-ibuprofen ester ${S_2}(x)$ versus dimensionless radial distance $x$ with numerical simulation results (bvp-4c) for the various Bodenstein number${B_0}$ using the Eqs. (11 ), and (13).

The derivation of a semi-analytical solution to incorporate the influence of ${B_0}$, directly proportional to the volumetric flow rate in the organic medium, on the efficiency of dynamic kinetic resolution. We obtained this solution to evaluate the performance of EMR. The Bodenstein number as noticed under the range of 8.68 to 34.72 (corresponding volumetric flow rate $50<F<200\,{\text{mL/min}}$)^13,14^, assuming the fixed values of ${\Phi ^2}=0.5,\,\Theta =3.27,\,\gamma =1$. When the ${B_0}=8.68$, the operating system at a flow rate below the $50\,{\text{mL/min}}$appeared inadequate due to unstable circumstances. The $F$ value indicates the volumetric flow rate of the bulk ester on the shell layer. As the Bodenstein number increases, there is a decrease in the volumetric flow rate, resulting in improved resolution. This phenomenon is caused by the dispersion of the substrate along the radial axis. An abrupt reduction in the initial substrate concentration was observed at the interface between the aqueous and organic phases, specifically at the boundary $x=2$ as portrayed in (Fig. 2**(a–b)**). The substrate and the immobilized enzyme exhibited rapid interaction within the intense reaction layer, referred to as the spongy matrix, in response to the efficient diffusion of substrate molecules through the membrane pore. With the increased value of the Bodenstein number ${B_0}$, the dimensionless concentration of (S)-ibuprofen ester increases, resulting in high conversion efficiency consisting of high purity in (S)-ester whereas (R)-ibuprofen ester decreases linearly against the radial direction, respectively (refer **Table. S1**,** Appendix F**,** Supplementary materials**).

**Fig. 3:**
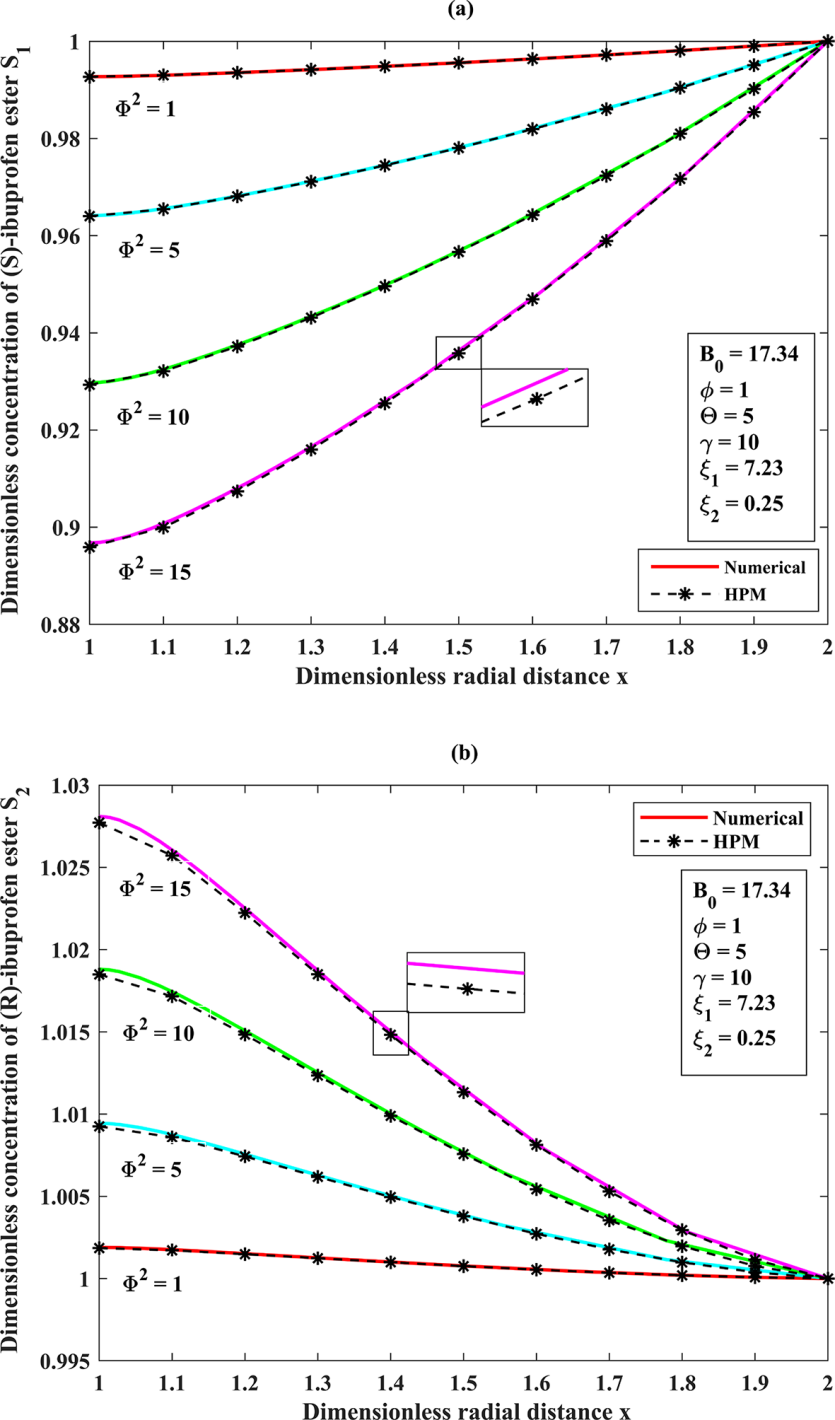
( **a** ) Comparison of semi-analytical (HPM) dimensionless concentration of (S)-ibuprofen ester versus dimensionless radial distance with numerical simulation results (bvp-4c) for the various diffusional restrictions using the Eqs. (10), and (12) **(b) **Comparison of semi-analytical (HPM) dimensionless concentration of (R)-ibuprofen ester versus dimensionless radial distance with numerical simulation results (bvp-4c) for the various diffusional restrictions using the Eqs. (11), and (13).

### A dimensionless concentration profile throughout the porous membrane support of the substrates (S)-ibuprofen and (R)-ibuprofen esters across the membrane matrix (1<x<2) for the effect of Thiele modulus ($\Phi^{2}$) under the steady-state assumptions

The Thiele modulus represents the ratio between the reaction rate happening on the surface of the membrane and the rate of molecular diffusion in a porous catalyst along the radial direction. It corresponds to the rate of a pseudo first-order reaction based on the rate of diffusion^5^. A Thiele modulus with a value greater than 1 indicates that the reaction is restricted by diffusion. Conversely, a Thiele modulus with a value less than 1 indicates that the reaction is limited by reaction kinetics against diffusion. From the (Fig. 3**(a–b)**), we can observe that the (S)-ibuprofen ester and (R)-ibuprofen ester will exhibit relatively uniform concentration profiles at the external surface of the catalyst, as diffusion effectively supplies substrates to the active sites when the Thiele modulus (${\Phi ^2}<<1$). In light of this, the concentration of (S)-ibuprofen ester and (R)-ibuprofen ester diminishes towards the centerline symmetry ($x=1$) of the catalyst particle when the Thiele modulus (${\Phi ^2}>>1$) that is, as the Thiele modulus ${\Phi ^2}$increases (enzyme loading is increased) leads to the substrate concentration decreasing across the spongy layer. Upon the values of ${\Phi ^2}=10,15$ when diffusional limits are present, it is possible that intraparticle diffusion restrictions are responsible for the substantial reduction in the formation of the pure (S)-enantiomer. As the diffusional restrictions ${\Phi ^2}$increases, the concentrations ${S_1}\left( x \right) \downarrow ,{S_2}\left( x \right) \uparrow$ at a fixed value of ${B_0}=17.34,\,\Theta =5,\,\gamma =10$(refer **Table. S2**,** Appendix F**,** Supplementary materials**).

**Fig. 4:**
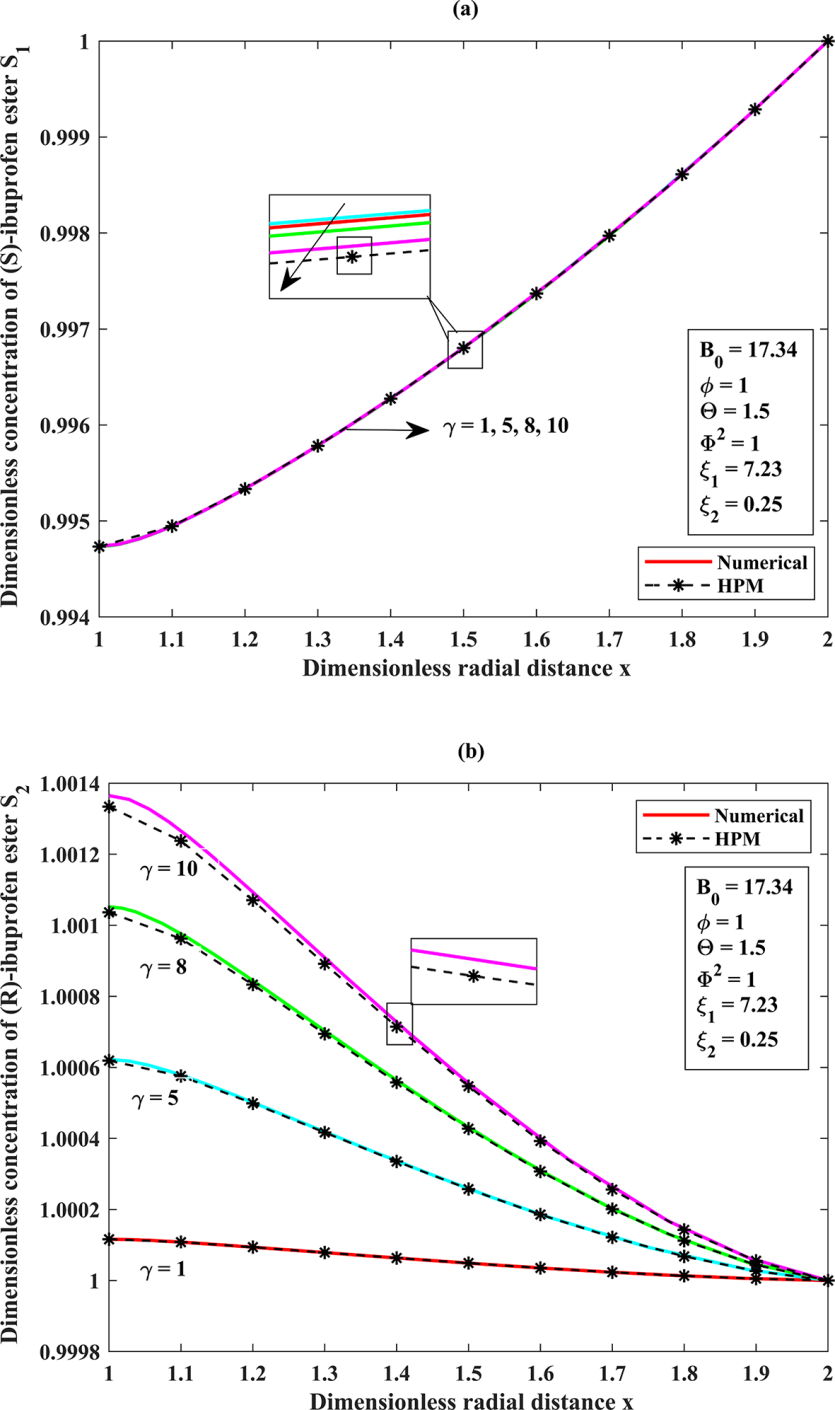
(**a**) Comparison of semi-analytical (HPM) dimensionless concentration of (S)-ibuprofen ester ${S_1}(x)$ versus dimensionless radial distance $x$ with numerical simulation results (bvp-4c) for the various diffusional restrictions ${\Phi ^2}$ using the Eqs. (10), and (12). (b) Comparison of semi-analytical (HPM) dimensionless concentration of (R)-ibuprofen ester${S_2}(x)$ versus dimensionless radial distance$x$ with numerical simulation results (bvp-4c) for the various diffusional restrictions${\Phi ^2}$ using the Eqs. ( 11 ), and (13).

**Fig. 5:**
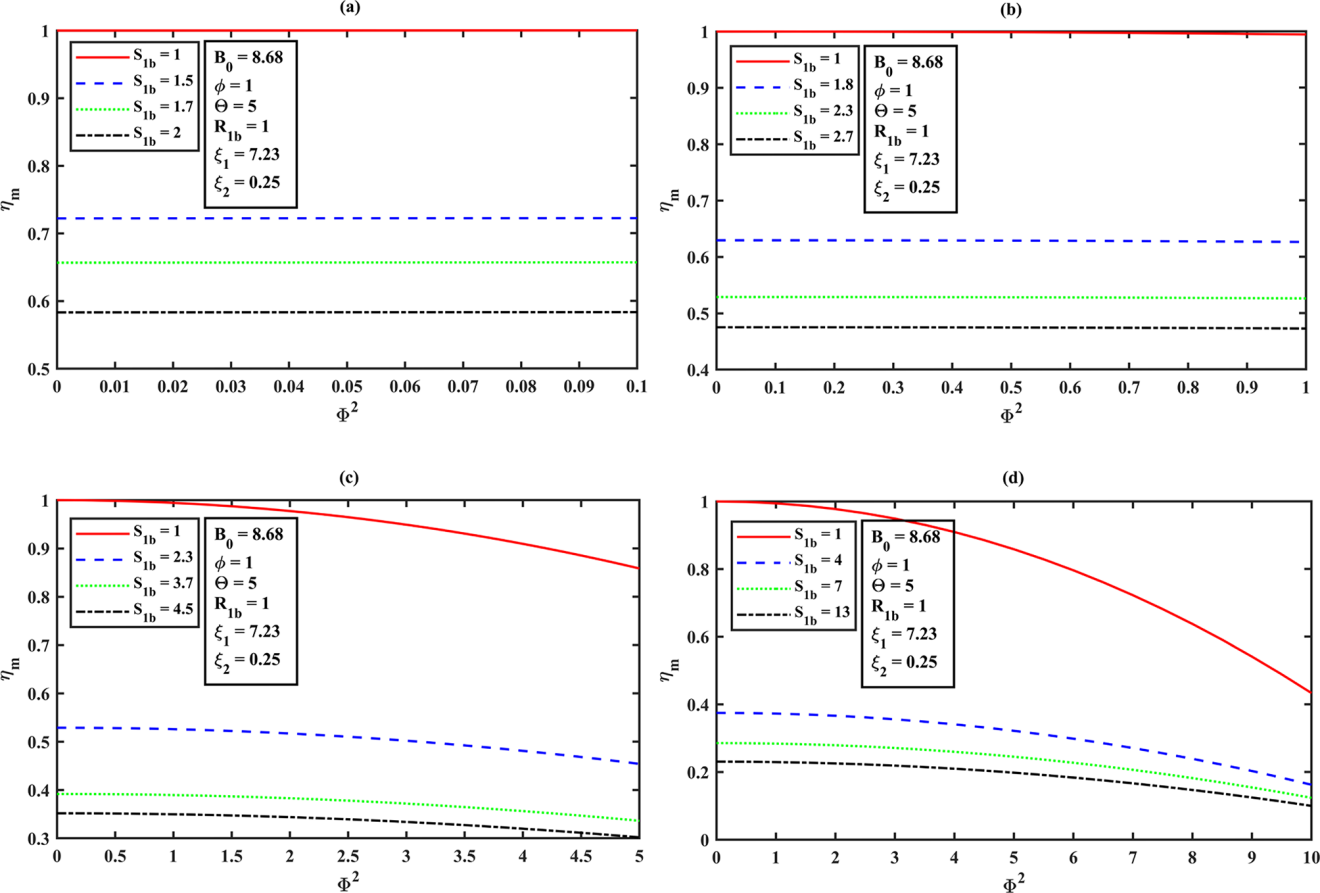
Dimensionless Thiele modulus against the mean integrated effectiveness factor () of the system for the various values of initial substrate concentration () in the bulk phase using the Eq. (22).

#### A dimensionless concentration profile throughout the porous membrane support of the substrates (S)-ibuprofen and (R)-ibuprofen esters across the membrane matrix ($1<x<2$ ) for the effect of racemization constant ($\gamma$ ) under the steady-state assumptions

For $\gamma <<1$, the concentration of (S)-ibuprofen ester is high near the inlet and low towards the outlet of the catalyst particle and shows a significant concentration gradient. The concentration of (R)-ester will remain less throughout the membrane since the conversion from (S)-ester to (R)-ibuprofen ester is minimum. For $\gamma \to 1$, the concentration profiles will be impacted by both intrinsic reactions and dynamics of racemization. For $\gamma>>1$, the concentration of (S)-ester and (R)-ester displays more uniform distribution across the membrane. As we can observed from the Fig. 4**(a–b)** and (refer **Table. S3**, **Appendix F**,** Supplementary materials**), the concentration of (S)-ester and (R)-ester is high as the racemization constant $\gamma$ varies for the distinct values along the radial direction.

**Fig. 6:**
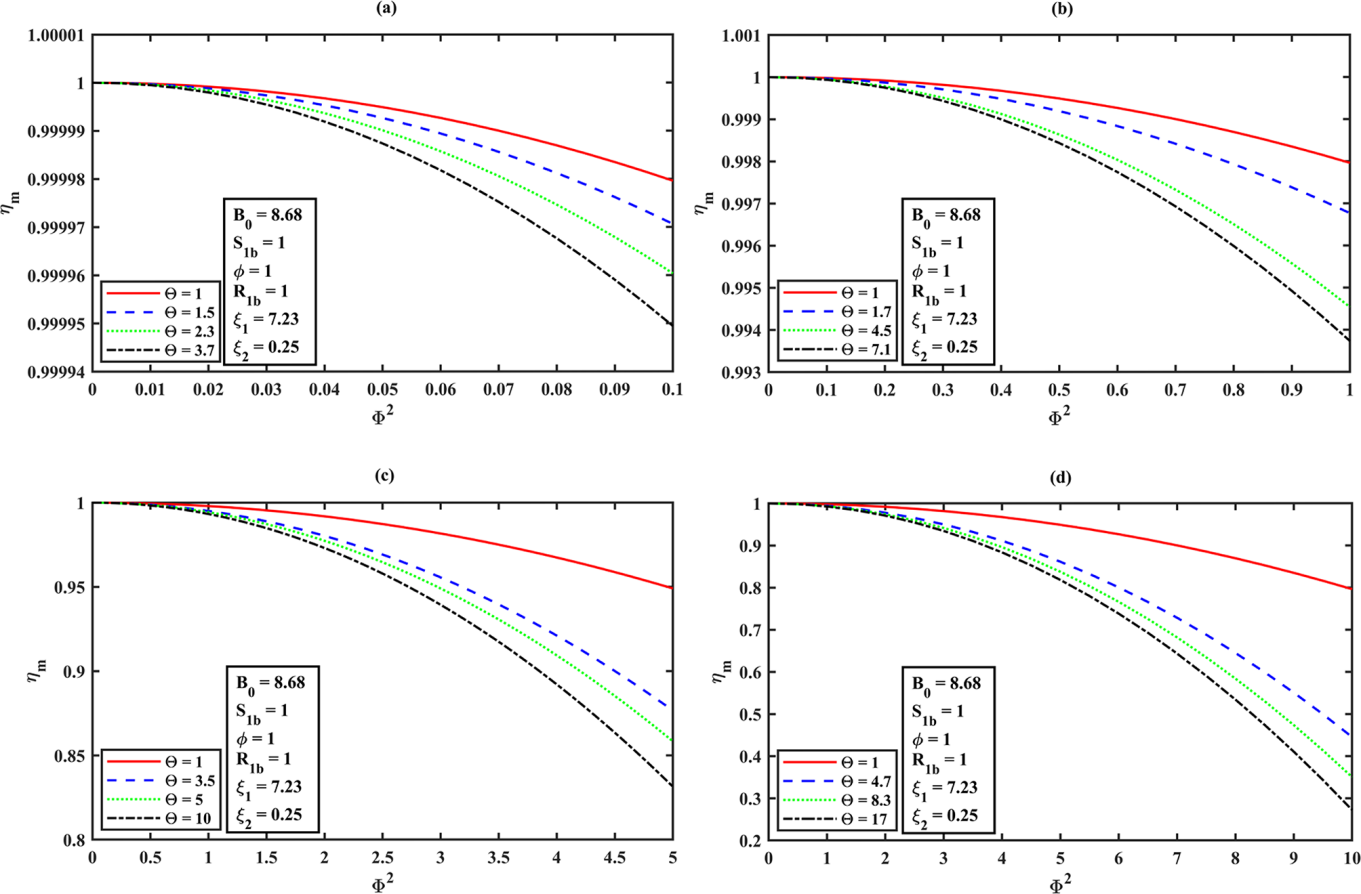
Dimensionless Thiele modulus against the mean integrated effectiveness factor () of the system for the various values of dimensionless Michaelis-Menten constant for (S)-ibuprofen ester () using the Eq. (22).

**Fig. 7:**
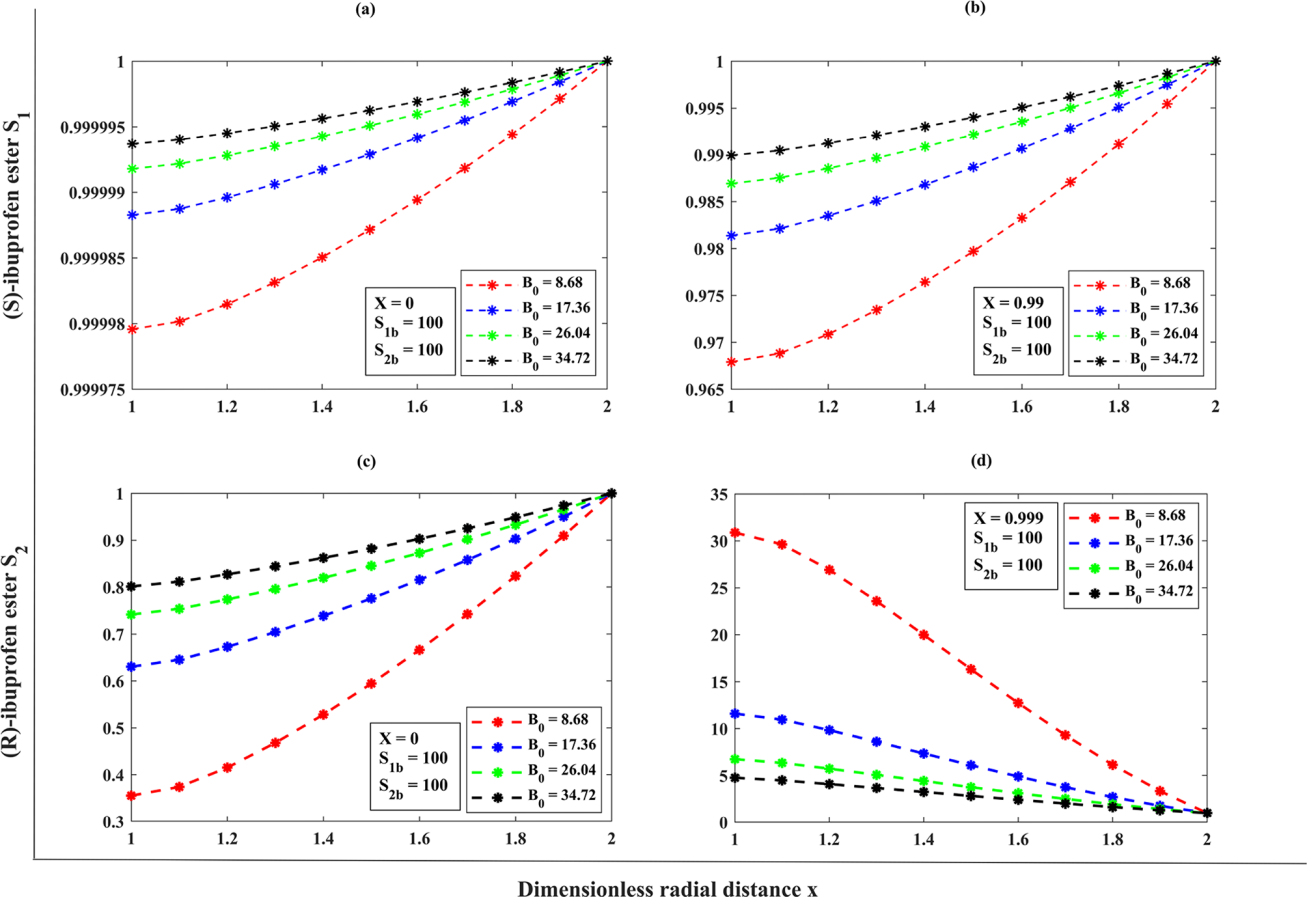
Dimensionless concentration of (S)-ibuprofen ester and (R)-ibuprofen ester against the radial distance by varying the distinct Bodenstein number for the conversion degree respectively, when the initial bulk concentration is 100, using the Eq. (17–18).

### Effectiveness factor ($\eta_{m}$ ) against the Thiele modulus ($\Phi^{2}$ ) effects to the system

In reference to Fig. 5**(a–d)** and from **Table 2**, we can see that for all values of Thiele modulus **(**${\Phi ^2}$**)**, ${\eta _m}$ decreases slightly as ${S_{1b}}$ increases. The Thiele modulus significantly affects the effectiveness factor (${\eta _m}$), with larger values typically resulting in all curves of effectiveness factor diminishing at low substrate concentrations. This observation reveals that a lower Thiele modulus and substrate concentration must be maintained to avoid diffusional restrictions and enzyme inhibition during (S)-ibuprofen ester hydrolysis. Furthermore, the impact of effectiveness factor (${\eta _m}$) as a function of Thiele modulus **(**${\Phi ^2}$**)** is as follows.

**Fig. 8:**
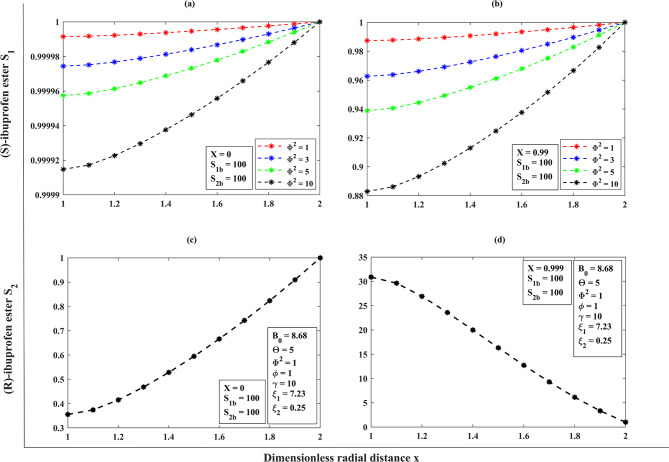
Dimensionless concentration of (S)-ibuprofen ester and (R)-ibuprofen ester against the radial distance by varying the distinct Thiele modulus for the conversion degree respectively, when the initial bulk concentration is 100 using the Eq. (17–18).

**Illustration 1.** When ($${\Phi ^2}<<\varepsilon ,\varepsilon =0.01,0.1,0.5,1 \Rightarrow {\eta _m} \to 1$$)

Assuming an enantioselective catalyst, it favors the formation of (S)-ibuprofen ester and the concentration throughout the particle is high. Besides, the concentration of (R)-ibuprofen ester is low or negligible according to the highly selective catalyst for (S)-ibuprofen ester for the distinct square of Thiele moduli (${\Phi ^2}$). Also, all the curves tend to achieve unity when transport resistances outside the enzyme are less relevant and instantly decline when kinetic input prevails (refer Fig. 6**(a–d)**, **Table 3**).

**Fig. 9:**
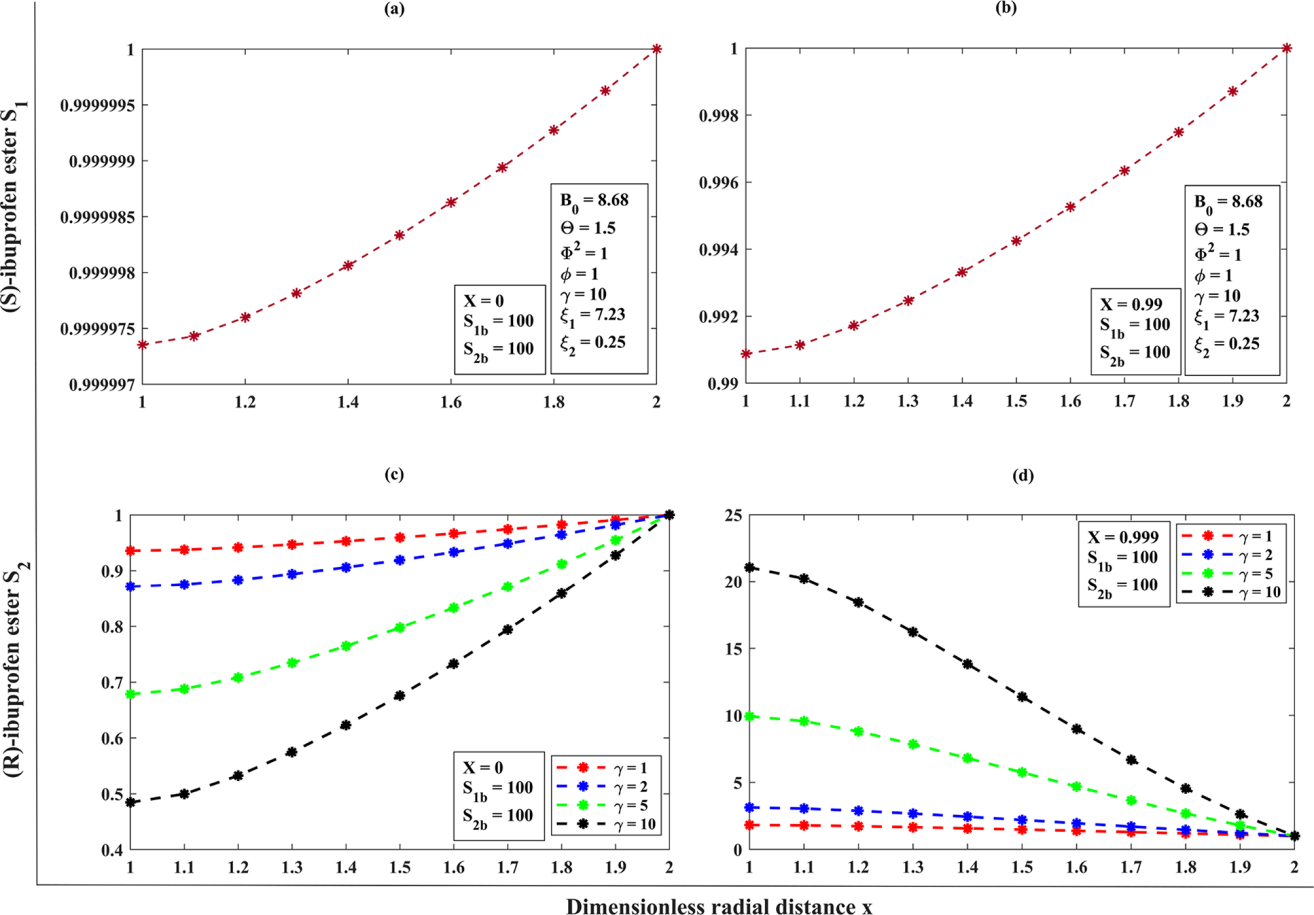
Dimensionless concentration of (S)-ibuprofen ester and (R)-ibuprofen ester against the radial distance by varying the distinct racemization constant for the conversion degree when the initial bulk concentration is 100 respectively, using the Eq. (17–18).

**Illustration 2.** When **(**$${\Phi ^2} \to \varepsilon ,\varepsilon =0.01,0.1,0.5,1 \Rightarrow {\eta _m}<<1$$**)**

In this scenario, the reaction and diffusion rates are equivalent and the concentration of (S)-ester is maximum near the particle surface and insignificant towards the dense layer. If the (R)-ester exists, it will show a gradient is lower in concentration, compared to the (S)-ester for an enantioselective reaction **(**refer Fig. 6**(a–d))**.

**Illustration 3.** When ($${\Phi ^2}>>\varepsilon ,\varepsilon =0.01,0.1,0.5,1 \Rightarrow {\eta _m} \to 0$$)

The concentration of (S)-ester is high at the surface and dropping significantly towards the centerline symmetry (dense layer). It is limited to the outer (spongy layer) region of the catalyst. On the other hand, (R)-ester concentration is very less. In this case, substrate rapidly consumed near the surface of the dense layer due to steep gradients and shows the lower concentrations internally for the various values of ${\Phi ^2}$which then implies, all the curves begin to deplete (i.e., ${\eta _m} \downarrow$) **(**refer Fig. 6**(a–d))**.

In reference to the Fig. 6**(a–d)** and from Table 3, for the higher values of $\Theta$, all the curves of mean integrated effectiveness factor ${\eta _m}$ decreases, as the square of Thiele modulus value ${\Phi ^2}$ increases. From Fig. 6**(a)**, we can observe that the system demonstrates a high level of effectiveness, irrespective of $\Theta$, as diffusion resistance has minimal impact on its performance. Figure 6**(b–d)** shows the diffusion limits become a crucial factor (the presence of stronger diffusion limitations), especially in systems where the affinity between the enzyme and substrate is low, as $\Theta$ is higher.

**Table 1:** Input kinetic parameters of DKR mass transport model and DKR reaction rate model in the hollow membrane reactor **[3**,**5**,**13**,**14]**.

Physical substances	Parameters	Values	Units
Base concentration	${s_{OH}}$	0–100	(mM)
Racemic substrate initial concentration	${s_{T0}}$	0.5–100	(mM)
Racemization reaction constant	${K_{rac}}$	0–5	(mM h^−1^)
Michaelis-Menten constant for (S)-enantiomer	${K_{mA}}$	10–50	(mM)
Maximum reaction rate	${v_{max}}$	0.001–80	(mM h^−1^)
Effective diffusivity	${D_{eff}}$	(1–10) × 10^−4^	(cm^2^/min)
Inner diameter	$a$	0.56	(mm)
Outer diameter	$c$	1.12	(mm)
Fiber length	*L*	43.2	(cm)
No. of fibers	*N*	65	–
Membrane porosity	${\alpha _p}$	0.8	–
Thiele modulus	${\Phi ^2}$	0–30	–
Bodenstein number	*B*_*0*_	8.68–34.72	–
Dimensionless racemization constant	$\gamma$	1–50	–
Dimensionless Michaelis-Menten constant	$\Theta$	0–100	–
Dimensionless substrate inhibition constant	${\xi _1}$	0–10	–
Dimensionless by-product inhibition constant	${\xi _2}$	0–10	–
Centerline symmetry radius	$\frac{b}{a}$	1	–
External fiber radius	$\frac{c}{a}$	2	–

**Table 2:** Mean integrated effectiveness factor ${\eta _m}$ for the system dimensionless concentration of (S)-ibuprofen ester (${S_1}$) versus the dimensionless Thiele modulus ${\Phi ^2}$ (a) 0.1 (b) 1 (c) 5 (d) 10 for the various values of dimensionless initial concentration in the bulk ${S_{1b}}$ using eq. (22), and ${B_0}=8.68,\phi =1,\Theta =5,{R_{1b}}=1,{\xi _1}=7.23,{\xi _2}=0.25$.

${\Phi ^2}=0.1$		${\Phi ^2}=1$		${\Phi ^2}=5$		${\Phi ^2}=10$	
${S_{1b}}$	${\eta _m}$	${S_{1b}}$	${\eta _m}$	${S_{1b}}$	${\eta _m}$	${S_{1b}}$	${\eta _m}$
1	0.999943	1	0.994334	1	0.858358	1	0.433430
1.5	0.722181	1.8	0.626062	2.3	0.454059	4	0.162536
1.7	0.656826	2.3	0.525988	3.7	0.336383	7	0.123837
2	0.583300	2.7	0.472616	4.5	0.302015	13	0.100022

**Table 3:** Mean integrated effectiveness factor ${\eta _m}$ for the system dimensionless concentration of (S)-ibuprofen ester (${S_1}$) against the dimensionless Thiele modulus ${\Phi ^2}$ (a) 0.1 (b) 1 (c) 5 (d) 10 for the distinct values of dimensionless Michaelis-Menten constant for (S)-ibuprofen ester $\Theta$ using eq. (22), and ${B_0}=8.68,\phi =1,{S_{1b}}={R_{1b}}=1,{\xi _1}=7.23,{\xi _2}=0.25$.

${\Phi ^2}=0.1$		${\Phi ^2}=1$		${\Phi ^2}=5$		${\Phi ^2}=10$	
$\Theta$	${\eta _m}$	$\Theta$	${\eta _m}$	$\Theta$	${\eta _m}$	$\Theta$	${\eta _m}$
1	0.999980	1	0.997960	1	0.949009	1	0.796035
1.5	0.999971	1.7	0.996766	3.5	0.876614	4.7	0.445295
2.3	0.999960	4.5	0.994538	5	0.858358	8.3	0.350161
3.7	0.999949	7.1	0.993732	10	0.831434	17	0.272273

**Table 4:** Comparison between the semi-analytical results for the dimensionless concentration of (S)-ibuprofen ester ${S_1}$ (eq. (10)) versus the numerical simulation for distinct values of Bodenstein number ${B_0}$ when ${\Phi ^2}=0.5,\phi =1,\Theta =3.27,\gamma =1,{\xi _1}=2,{\xi _2}=0.03$.

${B_0}=8.68$				${B_0}=34.72$		
*x*	HPM	Numerical (RK4M)	% of Error	HPM	Numerical (RK4M)	% of Error
1.0	0.983742	0.983745	0.000305	0.994948	0.994948	0
1.2	0.985243	0.985246	0.000304	0.995603	0.995603	0
1.4	0.988081	0.988083	0.000202	0.996495	0.996495	0
1.6	0.991548	0.991549	0.000101	0.997525	0.997525	0
1.8	0.995528	0.995528	0.000000	0.998693	0.998693	0
2.0	1.000000	1.000000	0.000000	1.000000	1.000000	0
Average % Error			0.000152	Average % Error		0

**Table 5:** Comparison between the semi-analytical results for the dimensionless concentration of (R)-ibuprofen ester ${S_2}$ (eq. (11)) versus the numerical simulation for distinct values of Bodenstein number ${B_0}$when ${\Phi ^2}=0.5,\phi =1,\Theta =3.27,\gamma =1,{\xi _1}=2,{\xi _2}=0.03$.

${B_0}=8.68$				${B_0}=34.72$		
*x*	HPM	Numerical (RK4M)	% of Error	HPM	Numerical (RK4M)	% of Error
1.0	1.000698	1.000699	0.000099	1.000056	1.000056	0
1.2	1.000583	1.000599	0.001599	1.000043	1.000043	0
1.4	1.000414	1.000426	0.001199	1.000028	1.000028	0
1.6	1.000244	1.000251	0.000699	1.000015	1.000015	0
1.8	1.000100	1.000102	0.000199	1.000005	1.000005	0
2.0	1.00000	1.000000	0	1.00000	1.00000	0
Average % Error			0.000633	Average % Error		0

**Table 6:** Comparison between the semi-analytical results for the dimensionless concentration of (S)-ibuprofen ester ${S_1}$ (eq. (10)) versus the numerical simulation for distinct values of Thiele moduli ${\Phi ^2}$ when ${B_0}=17.34,\phi =1,\Theta =5,\gamma =10,{\xi _1}=7.23,{\xi _2}=0.25$.

${\Phi ^2}=1$				${\Phi ^2}=10$		
*x*	HPM	Numerical (RK4M)	% of Error	HPM	Numerical (RK4M)	% of Error
1.0	0.992697	0.992702	0.000504	0.929310	0.929737	0.045948
1.2	0.993530	0.993533	0.000302	0.937188	0.937513	0.034678
1.4	0.994832	0.994835	0.000302	0.949605	0.949801	0.020640
1.6	0.996350	0.996351	0.000100	0.964212	0.964306	0.009749
1.8	0.998072	0.998072	0.000000	0.980986	0.981014	0.002854
2.0	1.000000	1.000000	0.000000	1.000000	1.000000	0.000000
Average % Error			0.000201	Average % Error		**0.018978**

**Table 7:** Comparison between the semi-analytical results for the dimensionless concentration of (R)-ibuprofen ester ${S_2}$ (eq. (11)) versus the numerical simulation for distinct values of Thiele moduli ${\Phi ^2}$when ${B_0}=17.34,\phi =1,\Theta =5,\gamma =10,{\xi _1}=7.23,{\xi _2}=0.25$.

${\Phi ^2}=1$				${\Phi ^2}=10$		
*x*	HPM	Numerical (RK4M)	% of Error	HPM	Numerical (RK4M)	% of Error
1.0	1.001851	1.001895	0.004392	1.018490	1.018812	0.031615
1.2	1.001486	1.001517	0.003095	1.014835	1.015069	0.023058
1.4	1.000992	1.001009	0.001698	1.009894	1.010027	0.013169
1.6	1.000545	1.000552	0.000699	1.005427	1.005486	0.005868
1.8	1.000198	1.000200	0.000199	1.001968	1.001985	0.001696
2.0	1.000000	1.000000	0.000000	1.000000	1.000000	0.000000
Average % Error			0.001681	Average % Error		0.012568

**Table 8:** Comparison between the semi-analytical results for the dimensionless concentration of (S)-ibuprofen ester ${S_1}$ (eq. (10)) versus the numerical simulation for distinct values of racemization constant $\gamma$ when ${B_0}=17.34,\phi =1,\Theta =1.5,{\Phi ^2}=1,{\xi _1}=7.23,{\xi _2}=0.25$.

$\gamma =5$				$\gamma =10$		
*x*	HPM	Numerical (RK4M)	% of Error	HPM	Numerical (RK4M)	% of Error
1.0	0.994733	0.994734	0.000101	0.994733	0.994736	0.000302
1.2	0.995334	0.995335	0.000100	0.995334	0.995336	0.000201
1.4	0.996274	0.996275	0.000100	0.996274	0.996276	0.000201
1.6	0.997369	0.997369	0.000000	0.997369	0.997370	0.000100
1.8	0.998611	0.998611	0.000000	0.998611	0.998611	0.000000
2.0	1.000000	1.000000	0.000000	1.000000	1.000000	0. 000000
Average % Error			0.000050	Average % Error		0.000134

**Table 9:** Comparison between the semi-analytical results for the dimensionless concentration of (R)-ibuprofen ester ${S_2}$ (eq. (11)) versus the numerical simulation for distinct values of racemization constant $\gamma$ when ${B_0}=17.34,\phi =1,\Theta =1.5,{\Phi ^2}=1,{\xi _1}=7.23,{\xi _2}=0.25$.

$\gamma =5$				$\gamma =10$		
*x*	HPM	Numerical (RK4M)	% of Error	HPM	Numerical (RK4M)	% of Error
1.0	1.000619	1.000623	0.000399	1.001334	1.001365	0.003096
1.2	1.000499	1.000502	0.000299	1.001070	1.001093	0.002298
1.4	1.000335	1.000337	0.000199	1.000714	1.000727	0.001299
1.6	1.000186	1.000186	0.000000	1.000392	1.000398	0.000599
1.8	1.000068	1.000068	0.000000	1.000142	1.000144	0.000199
2.0	1.000000	1.000000	0.000000	1.000000	1.000000	0.000000
Average % Error			0.000149	Average % Error		0.001249

### Conversion efficiency and concentration profiles of (S)-ibuprofen ester and (R)-ibuprofen ester in the membrane reactor

When the degree of conversion is low, say (*X* ≈ 0), and for the increasing values of Bodenstein number, the concentration of (S)-ibuprofen ester increases as the substrate is converted near the reactor inlet, whereas the concentration of (R)-ester is very low (refer Fig. 7**(a–d))**,** also (see**,** Table. (S4–S5)**, **Appendix F**,** Supplementary materials**). When the conversion degree is high ($X \to 1$), the concentration of (S)-ester reaches a maximum, reflecting efficient conversion throughout the reactor, and the (R)-ester concentration remains low if the racemization is minimal. For a lower degree of conversion (*X* ≈ 0), with the different increasing values of Thiele modulus, the concentration of (S)-ibuprofen ester increases sharply near the surface but much lower in the interior, and the concentration of (R)-ester raises near the surface when the racemization occurs effectively (refer Fig. 8**(a–d))**,** also (see**,** Table. S6**, **Appendix F**,** Supplementary materials**). For a higher degree of conversion ($X \to 1$), the concentration of (S)-ester is very high near the catalytic surface but may not reach maximum throughout the particle, and the (R)-ester concentration is significant, when the racemization is high or selectively low. For a low conversion degree (*X* ≈ 0), and for the distinct racemization constant, the (S)-ester concentration increases gradually, pointing to significant racemization and the (R)-ester concentration increases noticeably, reflecting high racemization rates (refer Fig. 9**(a–d))**,** also (see**,** Table. S7**, **Appendix F**,** Supplementary materials**). For a high conversion degree ($X \to 1$), the concentration of (S)-ester is lower than in the other cases, as racemization continuously converts (S)- to (R)-ibuprofen ester, and the (R)-ester concentration is high, potentially equal to the amount if equilibrium is reached. Furthermore, we have provided the reference graphs (refer **Fig. (S1–S3) (a–d)**,** Appendix F**,** Supplementary materials**) when the initial bulk concentration is 10.

**Fig. 10:**
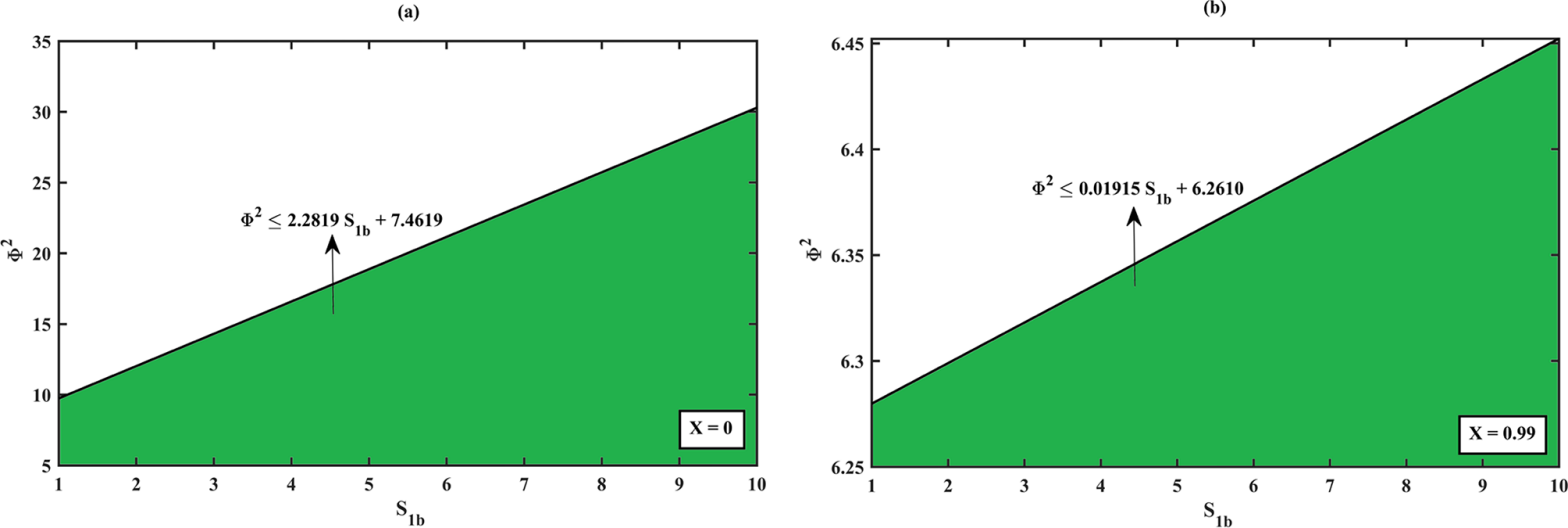
Limitation of the proposed solution (S)-ibuprofen ester using (HPM, Eq. (10)), at different degree of conversions *X= 0, 0.99* where valid region (green zone) and invalid region (white zone).

**Fig. 11:**
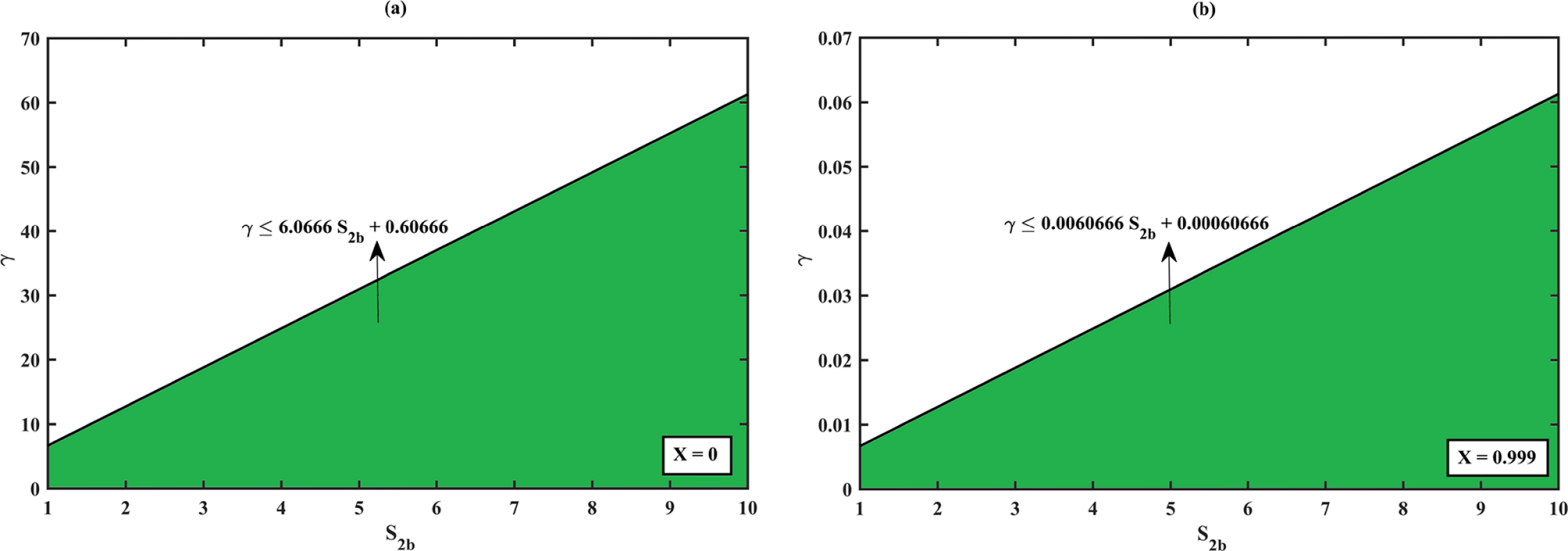
Limitation of the proposed solution (R)-ibuprofen ester using (HPM, Eq. (18)), at different degree of conversions *X= 0, 0.999* where valid region (green zone) and invalid region (white zone).

**Fig. 12:**
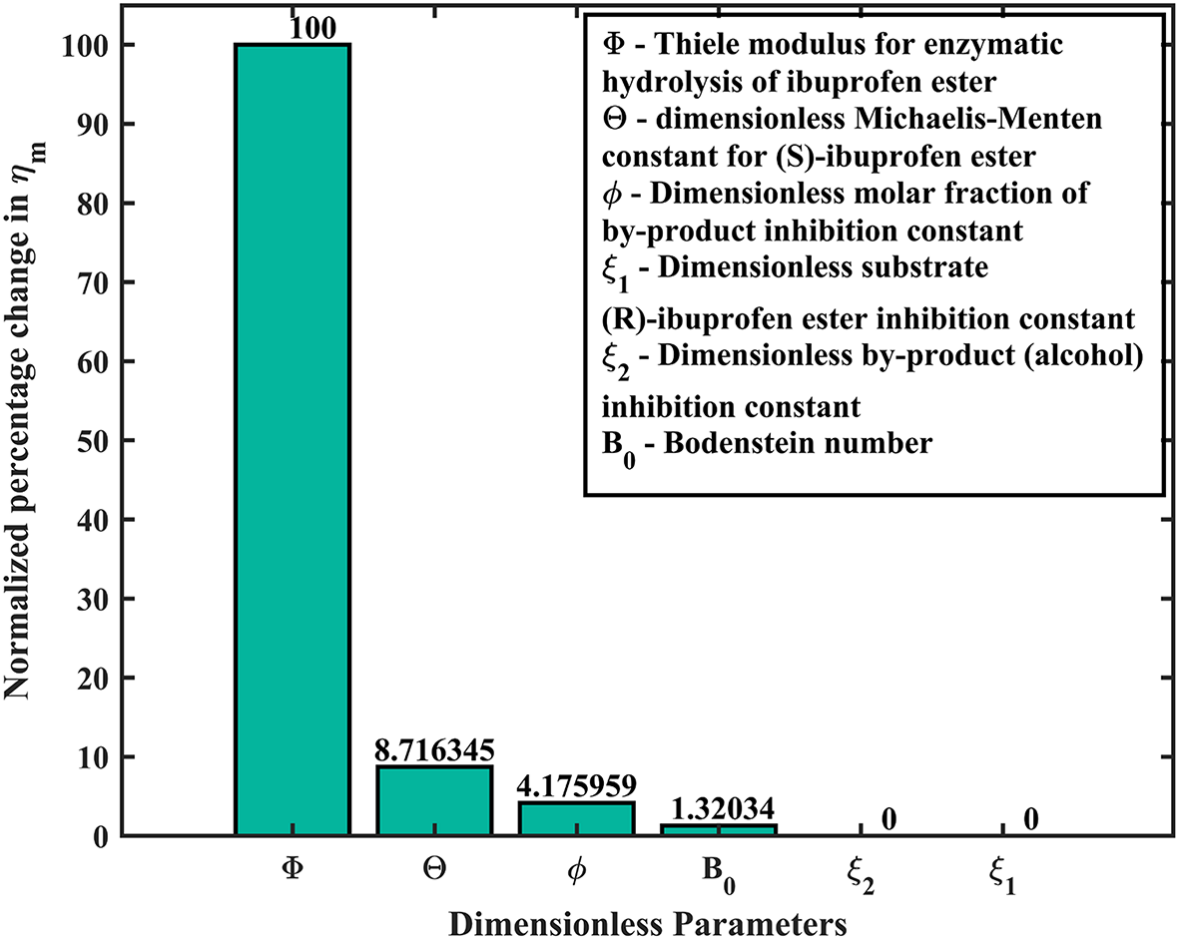
Normalized sensitivity analysis of the model parameters.

### Limitation of the proposed solution using (HPM, Eqs. (17–18)) for the dimensionless concentration of (S)- and (R)-ibuprofen ester

From Fig. 12**(a – b)**, we can observe that there is a connection between how well the proposed HPM solution works and the balance between reaction and diffusion rates, as indicated by the Thiele modulus ${\Phi ^2}$. The choice of Thiele modulus ${\Phi ^2}$ holds for the concentration of (S)-ibuprofen ester solution (HPM, Eq. (17)) to be valid/invalid when it satisfies the constraint equation ${\Phi ^2} \leqslant 2.2819\,{S_{1b}}+7.4619$ for all the values of ${S_{1b}}$, when the degree of conversion is $X\,=\,0$. If the choice of ${\Phi ^2}$exceeds (or does not satisfy the constraint) the solution diverges and falls into the invalid zone of the solution system (i.e., the tangent line indicates that for a given ${S_{1b}}$, the inequality ${\Phi ^2}$ must not exceed this value for the proposed solution to be valid). Moreover, the proposed HPM solution is applicable where the green zone (valid region) represents in the range of ${\Phi ^2}$ and ${S_{1b}}$. Within this region, the reaction kinetics and diffusion limitations are balanced so that the model assumptions hold true. Analogous to the above scenario, for all values of ${S_{1b}}$, when the degree of conversion is $X\,=\,0.99$, the choice of ${\Phi ^2}$ holds for the solution to be valid if it satisfies the constraint ${\Phi ^2} \leqslant 0.01915\,{S_{1b}}+6.261$. Furthermore, the valid zone indicates that for the proposed solution to remain valid at a high conversion rate, the Thiele modulus must be significantly lower. This means that when the conversion rate is high, the reaction is more diffusion-limited, and the catalyst needs to be used in a way that carefully balances the assumptions of the HPM. The invalid region indicates where the Thiele modulus exceeds the constraint, making the solution invalid. A similar observation on the limitation of (R)-ibuprofen ester solution using (HPM, Eq. (18)) can be noted as the choice of racemization constant $\gamma$ for the solution to be valid when it satisfies the following constraints $\gamma \leqslant 6.0666\,{S_{2b}}+0.60666$ with the degree of conversion $X\,=\,0$ and $\gamma \leqslant 0.0060666\,{S_{2b}}+0.00060666$ with the degree of conversion $X\,=\,0.999$ in reference to the Fig. 11**(a – b)**.

The higher degree of accuracy and the convergence for our proposed solution are observed when the series term is truncated to the third order (i.e., $O\left( {{p^3}} \right)$). Further, we can achieve the same level of accuracy in the solution, which can be obtained from the higher order series terms (i.e., $O\left( {{p^n}} \right);\,\,n>3$). We used a combination of a semi-analytical approach with the simulated numerical results. We solved the system of differential Eqs. (10)–(13) under the steady-state conditions using the bvp-4c algorithm **[27–31]** in MATLAB R2023b (https://in.mathworks.com/) **[32]** (see **Appendix C**,** E**). To exhibit the efficacy of our proposed method, we performed a comparison between the numerical results obtained for Eqs. (10)–(13) and our semi-analytical HPM results established from the expressions Eqs. (14)–(15) (see **Figs. (2–4) (a–b)**). Furthermore, we have included an error percentage that is referenced in the **Tables (4–9)**. The average relative error for the substrate concentrations between our semi-analytical results and numerical results is as follows: 0.018978%, 0.012568%, 0.001681%, 0.001249%, 0.000633%, 0.000201%, 0.000152%, 0.000149%, 0.000134%, 0.000050%, and 0.000%. Our findings indicate that there is no statistically significant difference between analytical and numerical solutions when considering a wide range of parameters such as the Thiele modulus values, Bodenstein number, and racemization constant.

### Normalized sensitivity analysis

The impact of each parameter using Eq. (22) for the mean integrated effectiveness factor (${\eta _m}$) is portrayed in the bar graph (see Fig. 12), which demonstrate the relationship between the parameters and their respective normalized values of sensitivities. The operating parameters $\Phi$ (Thiele modulus), $\Theta$ (dimensionless Michaelis-Menten constant for (S)-ibuprofen ester) have significant impacts, while the other factors, such has $\phi \,\,and\,{B_0}$ have the less moderate impacts and for the parameters ${\xi _1}\,{\text{and}}\,{\xi _2}$ has no direct impact on the mean integrated effectiveness factor ${\eta _m}$. The corresponding normalized percentage fluctuations are 100.0% for $\Phi$, 8.72% for $\Theta$, 4.18% for $\phi$, 1.32% for ${B_0}$, and 0% for ${\xi _1}\,{\text{and}}\,{\xi _2}$, respectively. The highest sensitivity ($\Phi$) indicates that the reaction rate is much faster compared to the diffusion rate, which can lead to diffusion limitations and thus lower effectiveness. The substantial sensitivity of the parameter ($\Theta$) Michaelis-Menten constant has a notable impact on the effectiveness factor ${\eta _m}$ which is lesser as compared to Thiele modulus, leads to reflecting the enzyme’s affinity for the substrate (S)-ibuprofen ester and its importance in the enzymatic reaction. The parameter ($\phi$) indicates the role of dimensionless molar fraction of by-product inhibition constant in the reaction system as it accounts for the inhibitory effect of by-product formed during the reaction. The Bodenstein number (${B_0}$) has a minor influence on ${\eta _m}$, indicates that mass transport effects are less critical compared to other parameters. The dimensionless by-product (alcohol) inhibition constant (${\xi _2}$) and the dimensionless substrate (R)-ibuprofen ester inhibition constant (${\xi _1}$) appear to have no effect under the given conditions, indicating that these specific inhibitory effects do not significantly alter the system. From this analysis, we observe that the solution curves demonstrate the overall effectiveness factor ${\eta _m}$, diminishes as the Thiele modulus increases (refer **Figs. (5–6) (a–d)**) for the various values of initial concentration of substrate in the bulk and the dimensionless Michaelis-Menten constant respectively.

## Conclusion

The semi-analytical solution using the effective method HPM is derived for the concentration profiles. We have analyzed the concentration profiles against Bodenstein number, Theile modulus, and racemization constant along the dimensionless radial axis using HPM. The dynamic performance of the mean integrated effectiveness factor is derived using HPM to understand the nature of the kinetic reaction system. The performance of normalized sensitivities is calculated to know the effective parameter amongst all operating parameters in our present model. We have contrasted the significant conversions of the (S)-ibuprofen ester and the (R)-ibuprofen ester with respect to various major parameters that were analyzed in order to check their behavior of the reaction kinetics, racemization, and the effectiveness of the catalytic process within the membrane reactor system. The MIEF against the Thiele modulus impact and the initial substrate concentration are obtained. The limitation of valid/invalid zone and the tangential behavior were opted for the system’s model with the different degree of conversions under the combination of bulk concentrations and the Thiele modulus effects. Our proposed semi-analytical technique was compared to BVP-4c numerical simulation results and have achieved a satisfactory level of agreement. Moreover, the semi-analytical solution that we have provided can assist in the design of membrane reactors by providing insights into the effects of various parameters on the separation process. This can lead to the development of more efficient and cost-effective industrial processes for the production of racemic ibuprofen ester. The ability to predict transport phenomena and reaction kinetics lays the groundwork for studying a wide range of enzyme-catalyzed reactions in membrane bioreactors. We can adapt the mathematical model beyond bench scale to evaluate industrial reactor designs, enabling data-driven optimization of pharmaceutical manufacturing processes.

## Electronic supplementary material

Below is the link to the electronic supplementary material.

Supplementary Material 1

## Data Availability

The data that support the findings of this study are available from the corresponding author upon reasonable request.

## Abbreviations

HPM: Homotopy perturbation method
DKR: Dynamic kinetic resolution of racemic ibuprofen esters
MIEF: Mean integrated effectiveness factor
EMR: Enzymatic membrane reactor
F: Volumetric flow rate of substrate stream (mL min^−1)^
N: Number of hollow fibers
D_eff_: effective diffusion coefficient for ibuprofen ester (cm^2^ min^−1^)
a, b, c: fiber internal/dense layer/external radius respectively (mm)
b: effective diffusion coefficient of PG (cm^2^ s^−1^)
*[E]*: enzyme concentration (g/L)
L: Effective length of the hollow fiber (cm)
S, S*: substrate (S)-ester molecule, substrate (R)-ester molecule
I: Inhibitor (alcohol) molecule
s_A_: Concentration of (S)-ibuprofen ester (mmol L^−1^)
s_B_: Concentration of (R)-ibuprofen ester (mmol L^−1^)
s_A0_, and s_B0_: Initial concentration of (S)- ibuprofen ester and (R)-ibuprofen ester respectively (mmol L^−1^)
s_T0_: Initial concentration of racemic ibuprofen ester (mmol L^−1^)
s_i_: substrate concentration of racemic ibuprofen ester (mmol L^−1^)
s_OH_: concentration of base catalyst (trioctylamine) (mmol L^−1^)
s_I_: substrate concentration of racemic ibuprofen ester (mmol L^−1^)
k_cat_: Hydrolysis reaction constant (mmol L^−1^)
K_rac_: racemization rate constant (mmol L^−1^ h^−1^)
K_m_: Michaelis-Menten constant for racemic ibuprofen ester (mmol L^−1^)
K_mA_, K_mB_: Michaelis-Menten constant for (S)- and (R)-ibuprofen ester (mmol L^−1^)
K_nI1_, K_nI2 _: Non-competitive alcohol inhibition constant (mmol L^−1^)
K_uS1_, K_uS2_: Uncompetitive substrate ester inhibition constant (mmol L^−1^)
v: Reaction rate for hydrolysis of ibuprofen ester (mmol L^−1^ h^−1^)
vmax: Maximum reaction rate for hydrolysis of ibuprofen ester (mmol L^−1^ h^−1^)
v_A_: Reaction rate of DKR of (S)-ibuprofen ester (mmol L^−1^ h^−1^)
v_B_: Reaction rate of DKR of (R)-ibuprofen ester (mmol L^−1^ h^−1^)
veq: Dynamic equilibrium rate (mmol L^−1^ h^−1^)
u(r): Radial flow velocity of bulk substrate at the shell side of the reactor (cm s^−1^)
r: fiber radius particle (mm)
t: reaction time (min)
