# Supplementary material for: Theoretical analysis of lipase-enzymatic DKR model for racemic (R)- and (S)- ibuprofen ester in a hollow cylindrical membrane bioreactor
Source: Sci Rep. 2025 Feb 15;15:5573. doi: 10.1038/s41598-025-89583-z (PMC11830055; doi:10.1038/s41598-025-89583-z)
Supplement: Supplementary file 1 — Supplementary Material 1 [file 41598_2025_89583_MOESM1_ESM.doc]

# Supplementary materials:

**Appendix A: Basic concept of homotopy perturbation method (HPM).**

In this study, we illustrate our problem by employing the homotopy perturbation method. We consider the following nonlinear differential equations by using the basic idea of this new method.

$A\left(u\right)-f\left(r\right)=0, r\in\Omega,$ (A.1)

And the associated boundary condition is

$B\left(u, \frac{\partial u}{\partial n}\right)=0, r\in\Gamma,$ (A.2)

Where $A$is a general differential operator, $B$is a boundary operator, $f\left(r\right)$is a known analytic function,$\Gamma$is the boundary of the domain $\Omega$ and$\frac{\partial}{\partial n}$represents differentiation along the normal drawn outwards from $\Omega$.


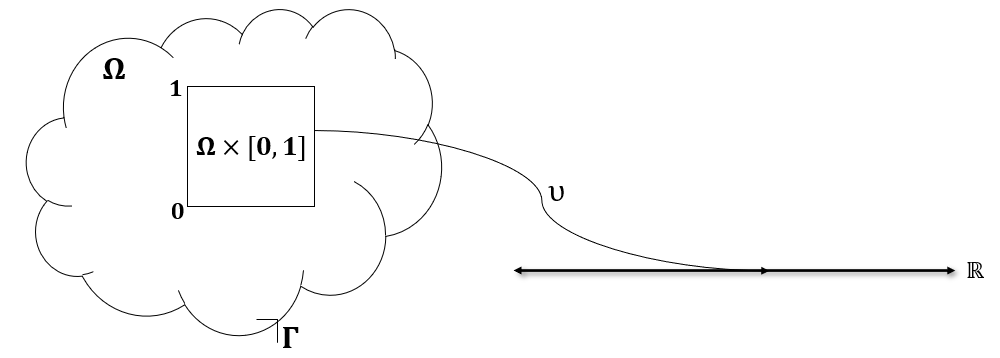


The operator generally divided into two parts $L$(linear) and $N$(nonlinear), Eq. (A.1) can be rewritten as follows:

$L\left(u\right)+N\left(u\right)-f\left(r\right)=0.$ (A.3)

By the homotopy perturbation technique, we construct a homotopy $\upsilon\left(r,p\right):\Omega\times\left[0,1\right]\to$ℝ which satisfies:

$H\left(\upsilon,p\right)=\left(1-p\right)\left[L\left(\upsilon\right)-L\left(u_{0}\right)\right]+p\left[A\left(\upsilon\right)-f\left(r\right)\right]=0, \forall p\in\left[0,1\right], \forall r\in\Omega,$ (A.4)

Where $p\in\left[0,1\right]$is an embedding (homotopy) parameter and $u_{0}$is an initial approximation which satisfies generally the boundary conditions of Eq. (A.1). Obviously, from this definition we will have:

$H\left(\upsilon,0\right)=L\left(\upsilon\right)-L\left(u_{0}\right)=0, H\left(\upsilon,1\right)=A\left(\upsilon\right)-f\left(r\right)=0$. (A.5)

The changing process of $p$from zero to unity is just that of $\upsilon\left(r,p\right)$from $u_{0}\left(r\right)$to$u\left(r\right)$. In topology, this is called deformation, and $L\left(\upsilon\right)-L\left(u_{0}\right)$&$A\left(\upsilon\right)-f\left(r\right)$ are called homotopy. The embedding parameter monotonically increases from zero to unit as the trivial problem $L\left(\upsilon\right)-L\left(u_{0}\right)=0$is continuously deformed to the original problem $A\left(\upsilon\right)-f\left(r\right)=0$. According to HPM, we can first use the embedding parameter$p$as a “small parameter”, and assume that the solution of Eq. (A.4) can be written as a power series in$p$.

$\upsilon=\upsilon_{0}+p\upsilon_{1}+p^{2}\upsilon_{2}+p^{3}\upsilon_{3}+\cdots$ (A.6)

Setting up $p=1$, the resulting approximate solution of (A.1) is as follows:

$u=\lim_{p\to1} \upsilon=\upsilon_{0}+\upsilon_{1}+\upsilon_{2}+\cdots$ (A.7)

**Appendix B: Analytical expressions for the dimensionless concentration of (R)- and (S)-ibuprofen esters using HPM technique.**

The dimensionless form of a governing nonlinear differential equations is:

, (B.1)

$\frac{d^{2}S_{2}}{dx^{2}}+\frac{1}{x}\left(1+B_{0}\right)\frac{dS_{2}}{dx}=\gamma\left(\frac{S_{1}-S_{2}}{S_{1}+S_{2}}\right)$, (B.2)

with the associated boundary conditions are:

$\frac{dS_{1}\left(x\right)}{dx}=\frac{dS_{2}\left(x\right)}{dx}=0 \text{at} x=1$, (B.3)

$S_{1}\left(x\right)$≈$S_{2}\left(x\right)$≈$1 \text{at } x=2$. (B.4)

To begin with HPM approach, we define a homotopy $\zeta\left(x,p\right),\upsilon\left(x,p\right):\Omega\times\left[0,1\right]\to$ℝ for the Eq. (B.1) and Eq. (B.2). Therefore, using the Eq. (A.4) we have,

, and $L\left(\upsilon\right)-L\left(S_{2_{0}}\right)+p L\left(S_{2_{0}}\right)+p \left[\gamma\left(\frac{\zeta-\upsilon}{\zeta+\upsilon}\right)\right]=0$. (B.5)

Here, $L=\frac{d^{2}}{dx^{2}}+\frac{1}{x}\left(1+B_{0}\right)\frac{d}{dx}$, is the linear operator, $p\in\left[0,1\right]$is an embedding parameter and we assume that subject to boundary conditions Eq. (B.3) and Eq. (B.4) the initial guess approximations of Eq. (B.1) and Eq. (B.2) are$S_{1_{0}}\left(x\right)=1, S_{2_{0}}\left(x\right)=1$. Assuming that the solution of (B.5) can be expressed as a power series in $p$, i.e.,

$\zeta\left(x,p\right)=\zeta_{0}\left(x\right)+p \zeta_{1}\left(x\right)+p^{2}\zeta_{2}\left(x\right)+\cdots$, and $\upsilon\left(x,p\right)=\upsilon_{0}\left(x\right)+p \upsilon_{1}\left(x\right)+p^{2}\upsilon_{2}\left(x\right)+\cdots$ (B.6)

Where $\zeta_{i}'s \text{and} \upsilon_{i}'s$are independent of $p$. Substituting Eq. (B.6) into Eq. (B.5), and resembling the equal powers of $p$give rise to a set of problems that we will now specify and solve in the succeeding sections.

**The Zeroth-order Problem (equating the coefficient of** $p^{0}$**):**

The differential equation of the zeroth-order problem is

$L\left(\zeta_{0}\right)-L\left(S_{1_{0}}\right)=0$ and $L\left(\upsilon_{0}\right)-L\left(S_{2_{0}}\right)=0$, i.e.,

$\frac{d^{2}\zeta_{0}\left(x\right)}{dx^{2}}+\frac{1}{x}\left(1+B_{0}\right)\frac{d\zeta_{0}\left(x\right)}{dx}=0$, and $\frac{d^{2}\upsilon_{0}\left(x\right)}{dx^{2}}+\frac{1}{x}\left(1+B_{0}\right)\frac{d\upsilon_{0}\left(x\right)}{dx}=0$,

Subject to the given boundary conditions

$\frac{d\zeta_{0}\left(1\right)}{dx}=\frac{d\upsilon_{0}\left(1\right)}{dx}=0$, and $\zeta_{0}\left(2\right)=\upsilon_{0}\left(2\right)=1$.

Since, $L$ is a linear operator, therefore the solution of the zeroth-order problem is $\zeta_{0}\left(x\right)=1, \upsilon_{0}\left(x\right)=1$.

**The First-order Problem (equating the coefficient of** $p^{1}$**):**

The differential equation of the first-order problem is

, and $\frac{d^{2}\upsilon_{1}\left(x\right)}{dx^{2}}+\frac{1}{x}\left(1+B_{0}\right)\frac{d\upsilon_{1}\left(x\right)}{dx}=0$,

Subject to the boundary conditions

$\frac{d\zeta_{1}\left(1\right)}{dx}=\frac{d\upsilon_{1}\left(1\right)}{dx}=0$, and $\zeta_{1}\left(2\right)=\upsilon_{1}\left(2\right)=0$.

Therefore, the solution of the first-order problem is

.

**The Second-order Problem (equating the coefficient of** $p^{2}$**):**

The differential equation of the first-order problem is

, and ,

Subject to the boundary conditions

$\frac{d\zeta_{2}\left(1\right)}{dx}=\frac{d\upsilon_{2}\left(1\right)}{dx}=0$, and $\zeta_{2}\left(2\right)=\upsilon_{2}\left(2\right)=0$,

Therefore, the solution of the first-order problem is

Therefore, collecting all these results we gain the approximate semi-analytical expressions for the dimensionless concentration of (R)- and (S)- ibuprofen esters using the homotopy perturbation method of the problem up to 2^nd^ order and the problem accuracy could be greatly improved by enhancing the order higher.

$$\begin{matrix} S_{1}\left(x\right)=\lim_{p\to1} \zeta\left(x,p\right)= \zeta_{0}\left(x\right)+ \zeta_{1}\left(x\right)+\zeta_{2}\left(x\right)+\cdots\text{and} \\ S_{2}\left(x\right)=\lim_{p\to1} \upsilon\left(x,p\right)= \upsilon_{0}\left(x\right)+ \upsilon_{1}\left(x\right)+\upsilon_{2}\left(x\right)+\cdots\end{matrix}$$

Therefore, the solution expression for the dimensionless concentration of (R)-ibuprofen ester is

And the solution expression for the dimensionless concentration of (S)-ibuprofen ester is

**Appendix C: Numerical solution for the dimensionless concentration of (R)- and (S)-ibuprofen esters using 3-stage Lobatto IIIA (Implicit Runge-Kutta method) along with shooting technique (BVP4C scheme).**

The bvp4c solver in MATLAB R2023b **[32]** is implemented for solving the nonlinear ordinary differential equations that govern the dimensionless concentration of (R)- and (S)- ibuprofen esters, subject to given boundary conditions as described by the equations (B.1) - (B.4). The successive step is to alter the Eq. (C.1) into a set of first-order ODEs by defining new variables,

$S_{1}=f_{1}, \frac{dS_{1}}{dx}=f_{2}, S_{2}=f_{3}, \frac{dS_{2}}{dx}=f_{4},$ (C.1)

Substituting in the Eq. (C.1), we get the subsequent arrangement of first-order equations:

, (C.2)

$\left.\begin{matrix} \frac{df_{3}}{dx}=f_{4} \\ \frac{df_{4}}{dx}=-\frac{1}{x}\left(1+B_{0}\right)f_{4}+\gamma\left(\frac{f_{1}-f_{3}}{f_{1}+f_{3}}\right) \end{matrix}\right\}$, (C.3)

Subsequently, the corresponding boundary conditions are

$f_{2}\left(1\right)=0, f_{4}\left(1\right)=0, f_{1}\left(2\right)=1, f_{3}\left(2\right)=1$. (C.4)

Eqs. (C.2) - (C.4) may be subjected to numerical integration until an endpoint is reached, hence enabling the determination of an initial value problem. Using MATLAB R2023b, we can run this program with a step size of 0.01, determining the range from 1 to 2 and return.


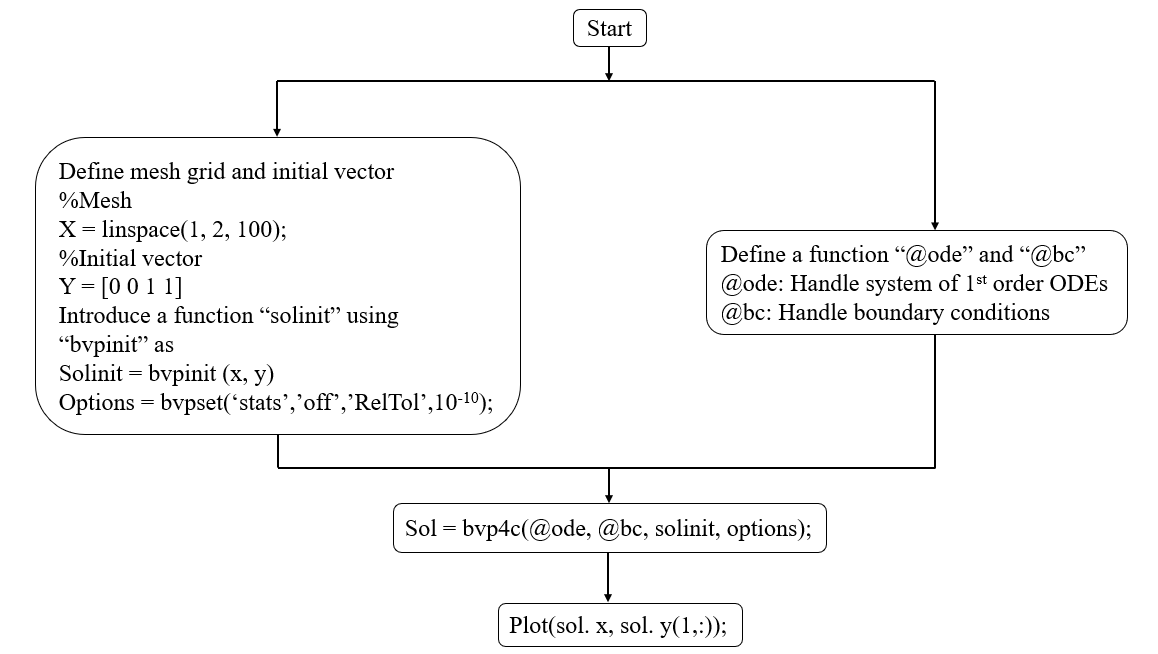


**Flowchart of BVP4C MATLAB code**

**Appendix D: Simplification of Robin boundary conditions (linear combination of Dirichlet and Neumann conditions) for the Eq. (13).**

From the external fiber radius Eq. (7), after converting into dimensionless state, the boundary condition at $x=2$ becomes,

$\frac{dS_{i}\left(x\right)}{dx}=\frac{B_{0}}{x} \left(1-S_{i}\left(x\right)\right), \forall i=1,2$,

On solving the above differential equations,

$$\begin{matrix} \Rightarrow\int\frac{dS_{i}}{\left(1-S_{i}\right)}=\int\frac{B_{0}}{x} dx, \forall i=1,2 \\ \Rightarrow-\ln\left(1-S_{i}\right)=B_{0}\ln\left(x\right), \forall i=1,2 \\ \Rightarrow\ln\left(1-S_{i}\right)^{-1}=\ln\left(x^{B_{0}}\right), \forall i=1,2 \\ \Rightarrow\frac{1}{1-S_{i}}=x^{B_{0}}, \forall i=1,2 \\ \Rightarrow S_{i}\left(x\right)=1-\frac{1}{x^{B_{0}}}, \forall i=1,2 \end{matrix}$$

Therefore, at $x=2,$we have,

$$S_{i}\left(x\right)=1-\frac{1}{2^{B_{0}}}, \forall i=1,2.$$

Now, as $B_{0}>>10\Rightarrow\frac{1}{2^{B_{0}}}\to0$,

Hence, the second boundary conditions become,

$$S_{i}\left(x\right)\approx1, \forall i=1,2.$$

**Appendix E: MATLAB R2023b simulation codes:**

1. **BVP-4C MATLAB syntax**

%%%%% BVP-4C (Runge-Kutta 4th order method) %%%%%

clear

close all

clc

global b0 Phi phi th xi1 xi2 ga

infinite = 2;

%%%% varying Bodenstein number %%%%%

Phi=sqrt(0.5); phi=1; th=3.27; ga=1; xi1=2; xi2=0.03;

b0va=[8.68 17.36 24.06 34.72];

%%%%% varying Thiele Modulus values %%%%%%%%

% b0=17.34; Phi=1; phi=1; th=5; ga=10; xi1=7.23; xi2=0.25;

% Phiva=[1 sqrt(5) sqrt(10) sqrt(15)];

%%%%%% Varying racemization constant %%%%%

%

% b0=17.34; Phi=10; phi=1; th=5; xi1=3.5; xi2=5;

% gava=[1 2 5 8 10];

for j=1:4

b0=b0va(j);

solinit = bvpinit(linspace(1,infinite,10),[0 0 1 1]);

sol = bvp4c(@shootode,@shootbc,solinit);

eta = sol.x;

f = sol.y;

lines = {'r','b','g','k','m*','-yo','-ko'};

plot(eta,f(1,:),lines{j},'linewidth',1.5)

%xlabel('\bf Dimensionless radial distance x','FontSize',11,'FontWeight','bold');

%ylabel('\bf Dimensionless concentration of (R)-ibuprofen ester S_2','FontSize',11,'FontWeight','bold');

% eta_eval = 1;

% f_eval = deval(sol, eta_eval);

% fprintf('f=%.6f \n', f(3,:))

% legend( 'Numerical','','','','' );

hold on

end

function dydx = shootode(eta,f)

global b0 Phi phi th xi1 xi2 ga

dydx = [ f(2)

-(((1+b0).*f(2))./eta)+((Phi.^2).*f(1))./((1+(f(1)./th)).*(1+phi*xi2+f(3)*xi1))

f(4)

-(((1+b0).*f(4))./eta)+ga*((f(1)-f(3))./(f(1)+f(3)))

];

end

% function res = shootbc(fa,fb)

% global b0

% res = [fa(2)

% fa(4)

% fb(2)-(b0.*0.5.*(1-fb(1)))

% fb(4)-(b0.*0.5.*(1-fb(3)))

% ];

% end

function res = shootbc(fa,fb)

res = [fa(2)

fa(4)

fb(1)-1

fb(3)-1

];

end

1. **Homotopy perturbation method (HPM solutions) MATLAB syntax**

clc

clear

%close all

%%%%%% HPM solution %%%%%%%

%global b0 Phi Theta xi1 xi2 g r

x = (1:0.1:2);

%x = linspace(1,2);

%%%% varying Bodenstein number %%%%%

b0=8.68; Phi=sqrt(0.5); phi=1; g=1; Theta=3.27; xi1=2; xi2=0.03;

b0va=[8.68 17.36 24.06 34.72];

%%%%% varying Thiele Modulus values %%%%%%%%

% b0=17.34; Phi=1; g=10; Theta=5; xi1=7.23; xi2=0.25;

% Phiva=[sqrt(1.2) sqrt(5.8) sqrt(11.6) sqrt(17.2)];

% %Phiva=[1 sqrt(5) sqrt(10) sqrt(15)];

%

% %%%%%% Varying racemization constant %%%%%

%

% b0=17.34; Phi=1; g=10; Theta=1.5; xi1=7.23; xi2=0.25;

% gva=[1 5 8 10];

%%%%% Concentration of (S)-ibuprofen ester [S_1] profiles %%%%%%%%%%

figure

for j=1:4

b0=b0va(j);

%Phi=Phiva(j);

%g=gva(j);

S1 = 0.1e1 + (Phi .^ 2 .* Theta .* (x .^ 2 .* b0 - 4 .* b0 - 2 .^ (1 - b0) + 2 .* x .^ (-b0)) ./ b0 ./ (2 + b0) ./ (1 + Theta) ./ (phi .* xi2 + xi1 + 1)) ./ 0.2e1 + (Phi .^ 4 .* Theta .^ 3 .* ((-4 .* b0 .^ 3 - 24 .* b0 .^ 2 - 32 .* b0) .* x .^ (2 - b0) + ((-8 .* b0 .^ 2 - 16 .* b0 + 64) .* x .^ (-b0) - 4 .* x .^ 2 .* b0 .^ 3 - 8 .* x .^ 2 .* b0 .^ 2 + 32 .* x .^ 2 .* b0 + 40 .* b0 .^ 3 + 144 .* b0 .^ 2 - 96 .* b0 + 64) .* 2 .^ (-b0) + (-2 + b0) .* ((-8 .* b0 .^ 2 - 32 .* b0 + 32) .* x .^ (-b0) + (8 .* b0 + 32) .* 4 .^ (-b0) + ((x .^ 2 - 4) .* b0 + 2 .* x .^ 2 - 24) .* (x .^ 2 - 4) .* b0 .^ 2)) ./ b0 .^ 2 ./ (4 + b0) ./ (-2 + b0) ./ (2 + b0) .^ 2 ./ (1 + Theta) .^ 3 ./ (phi .* xi2 + xi1 + 1) .^ 2) ./ 0.8e1;

lines = {'--r*','--b*','--g*','--k*','--k*','--k*','--k*'};

plot(x,S1,lines{j},'linewidth',1)

% xlabel('\bf Dimensionless radial distance x','FontSize',11,'FontWeight','bold');

% ylabel('\bf Dimensionless concentration of (S)-ibuprofen ester S_1','FontSize',11,'FontWeight','bold');

% %legend( 'Numerical','','','','HPM' ,'','','','');

% %fprintf('%.6f \n', x, S1)

hold on

end

%%%%% Concentration of (R)-ibuprofen ester [S_2] profiles %%%%%%%%%%

figure

for j=1:4

b0=b0va(j);

%Phi=Phiva(j);

%g=gva(j);

S2 = 0.1e1 + g .* Phi .^ 2 .* Theta .* ((-(3 .* b0 .^ 2) - (4 .* b0) - (b0 .^ 3) ./ 0.2e1) .* x .^ (2 - b0) + ((-b0 .^ 2 - 2 .* b0 + 8) .* x .^ (-b0) - x .^ 2 .* (b0 .^ 3) ./ 0.2e1 - x .^ 2 .* (b0 .^ 2) + 0.4e1 .* x .^ 2 .* b0 + (5 .* b0 .^ 3) + (18 .* b0 .^ 2) - (12 .* b0) + 0.8e1) .* (2 .^ (-b0)) + (-2 + b0) .* ((-b0 .^ 2 - 4 .* b0 + 4) .* x .^ (-b0) + ((4 + b0) .* 4 .^ (-b0)) + ((x .^ 2 - 0.4e1) .* b0 + 0.2e1 .* x .^ 2 - 0.24e2) .* (x .^ 2 - 0.4e1) .* (b0 .^ 2) ./ 0.8e1)) ./ (b0 .^ 2) ./ (4 + b0) ./ (-2 + b0) ./ ((2 + b0) .^ 2) ./ (1 + Theta) ./ (phi .* xi2 + xi1 + 1) ./ 0.2e1;

lines = {'--r*','--b*','--g*','--k*','--k*','--k*','--k*'};

plot(x,S2,lines{j},'linewidth',1)

xlabel('\bf Dimensionless radial distance x','FontSize',11,'FontWeight','bold');

ylabel('\bf Dimensionless concentration of (S)-ibuprofen ester S_1','FontSize',11,'FontWeight','bold');

%legend( 'Numerical','','','','HPM' ,'','','','');

%fprintf('%.6f \n', x, S1)

hold on

end

%%%%%%%%%%%%%%%%% End %%%%%%%%%%%%%%%%%%%%%%%%

**Appendix F: Additional figures and tables for reference/review**

**List of Tables:**

**Table. S1** Dimensionless concentration of (S)-ibuprofen ester $S_{1}$ and (R)-ibuprofen ester $S_{2}$ (HPM, Eq. (10 – 13)) for the various values of Bodenstein number $B_{0}$ at $x=1$ and ().

| $B_{0}$ | $S_{1}$ | $B_{0}$ | $S_{2}$ |
| --- | --- | --- | --- |
| 8.68 | 0.983742 | 8.68 | 1.00070 |
| 17.34 | 0.990624 | 17.34 | 1.00021 |
| 24.06 | 0.992949 | 24.06 | 1.00010 |
| 34.72 | 0.994948 | 34.72 | 1.00006 |

**Table. S2** Dimensionless concentration of (S)-ibuprofen ester $S_{1}$ and (R)-ibuprofen ester $S_{2}$ (HPM, Eq. (10 – 13)) for the various values of Thiele modulus $\Phi^{2}$ at $x=1$ and ().

| $\Phi^{2}$ | $S_{1}$ | $\Phi^{2}$ | $S_{2}$ |
| --- | --- | --- | --- |
| 1 | 0.992697 | 1 | 1.00185 |
| 5 | 0.964006 | 5 | 1.00926 |
| 10 | 0.929310 | 10 | 1.01849 |
| 15 | 0.895913 | 15 | 1.02772 |

**Table. S3** Dimensionless concentration of (S)-ibuprofen ester $S_{1}$ and (R)-ibuprofen ester $S_{2}$ (HPM, Eq. (10 – 13)) for the various values of Thiele modulus $\gamma$ at $x=1$ and ().

| $\gamma$ | $S_{1}$ | $\gamma$ | $S_{2}$ |
| --- | --- | --- | --- |
| 1 | 0.994733 | 1 | 1.00012 |
| 5 | 0.994734 | 5 | 1.00062 |
| 8 | 0.994735 | 8 | 1.00104 |
| 10 | 0.994736 | 10 | 1.00133 |

**Table. S4** Concentration of (S)-ibuprofen ester $S_{1}$ (HPM, Eq. (17)) for various values of Bodenstein number $B_{0}$ at $x=1$, for the distinct values of initial bulk concentrations $S_{1b},S_{2b}$, and the conversion $X$with .

| $S_{1b}=S_{2b}=10, X=0$ | | $S_{1b}=S_{2b}=100, X=0$ | | $S_{1b}=S_{2b}=10, X=0.99$ | | $S_{1b}=S_{2b}=100, X=0.99$ | |
| --- | --- | --- | --- | --- | --- | --- | --- |
| $B_{0}$ | $S_{1}$ | $B_{0}$ | $S_{1}$ | $B_{0}$ | $S_{1}$ | $B_{0}$ | $S_{1}$ |
| 8.68 | 0.998482 | 8.68 | 0.999980 | 8.68 | 0.9094343 | 8.68 | 0.967905 |
| 17.34 | 0.999127 | 17.34 | 0.999988 | 17.34 | 0.943176 | 17.34 | 0.981357 |
| 26.04 | 0.999389 | 26.04 | 0.999992 | 26.04 | 0.959820 | 26.04 | 0.986922 |
| 34.72 | 0.999531 | 34.72 | 0.999994 | 34.72 | 0.968936 | 34.72 | 0.989930 |
| 54.13 | 0.999691 | 54.13 | 0.999996 | 54.13 | 0.979407 | 54.13 | 0.993353 |

**Table. S5** Concentration of (R)-ibuprofen ester $S_{2}$ (HPM, Eq. (18)) for various values of Bodenstein number $B_{0}$ at $x=1$, for the distinct values of initial bulk concentrations $S_{1b},S_{2b}$, and the conversion $X$with .

| $S_{1b}=S_{2b}=10, X=0$ | | $S_{1b}=S_{2b}=100, X=0$ | | $S_{1b}=S_{2b}=10, X=0.999$ | | $S_{1b}=S_{2b}=100, X=0.999$ | |
| --- | --- | --- | --- | --- | --- | --- | --- |
| $B_{0}$ | $S_{2}$ | $B_{0}$ | $S_{2}$ | $B_{0}$ | $S_{2}$ | $B_{0}$ | $S_{2}$ |
| 8.68 | 0.392352 | 8.68 | 0.355396 | 8.68 | 2710.66 | 8.68 | 30.8856 |
| 17.34 | 0.657138 | 17.34 | 0.630042 | 17.34 | 836.806 | 17.34 | 11.6186 |
| 26.04 | 0.762032 | 26.04 | 0.741605 | 26.04 | 401.159 | 26.04 | 6.74889 |
| 34.72 | 0.817742 | 34.72 | 0.801461 | 34.72 | 236.873 | 34.72 | 4.76695 |
| 54.13 | 0.880395 | 54.13 | 0.869256 | 54.13 | 105.279 | 54.13 | 3.01697 |

**Table. S6** Concentration of (S)-ibuprofen ester $S_{1}$ (HPM, Eq. (17)) for various values of Thiele moduli $\Phi^{2}$ at $x=1$, for the distinct values of initial bulk concentrations $S_{1b},S_{2b}$, and the conversion $X$with .

| $S_{1b}=S_{2b}=10, X=0$ | | $S_{1b}=S_{2b}=100, X=0$ | | $S_{1b}=S_{2b}=10, X=0.99$ | | $S_{1b}=S_{2b}=100, X=0.99$ | |
| --- | --- | --- | --- | --- | --- | --- | --- |
| $\Phi^{2}$ | $S_{1}$ | $\Phi^{2}$ | $S_{1}$ | $\Phi^{2}$ | $S_{1}$ | $\Phi^{2}$ | $S_{1}$ |
| 1 | 0.999412 | 1 | 0.999991 | 1 | 0.938196 | 1 | 0.987359 |
| 3 | 0.998239 | 3 | 0.999974 | 3 | 0.830424 | 3 | 0.962695 |
| 5 | 0.997067 | 5 | 0.999957 | 5 | 0.743766 | 5 | 0.938858 |
| 10 | 0.994144 | 10 | 0.999915 | 10 | 0.619492 | 10 | 0.882877 |
| 17 | 0.990072 | 17 | 0.999855 | 17 | 0.608268 | 17 | 0.813174 |

**Table. S7** Concentration of (R)-ibuprofen ester $S_{2}$ (HPM, Eq. (18)) for various values of racemization constant $\gamma$ at $x=1$, for the distinct values of initial bulk concentrations $S_{1b},S_{2b}$, and the conversion $X$with .

| $S_{1b}=S_{2b}=10, X=0$ | | $S_{1b}=S_{2b}=100, X=0$ | | $S_{1b}=S_{2b}=10, X=0.999$ | | $S_{1b}=S_{2b}=100, X=0.999$ | |
| --- | --- | --- | --- | --- | --- | --- | --- |
| $\gamma$ | $S_{2}$ | $\gamma$ | $S_{2}$ | $\gamma$ | $S_{2}$ | $\gamma$ | $S_{2}$ |
| 1 | 0.941400 | 1 | 0.935778 | 1 | 33.8742 | 1 | 1.82409 |
| 5 | 0.702188 | 7 | 0.549333 | 5 | 694.463 | 7 | 16.8695 |
| 8 | 0.517730 | 10 | 0.355396 | 8 | 1745.45 | 10 | 30.8856 |
| 10 | 0.392352 | 13 | 0.160983 | 10 | 2710.66 | 13 | 49.2307 |
| 15 | 0.070491 | 15 | 0.031110 | 15 | 6049.58 | 15 | 63.8657 |

**List of Figures:**





**Fig. S1 Dimensionless concentration of (S)-ibuprofen ester $S_{1}(x)$ and (R)-ibuprofen ester $S_{2}(x)$ against the radial distance $x$ by varying the distinct Bodenstein number $B_{0}$ for the conversion degree $X=0,0.99,0.999$ respectively, when the initial bulk concentration is 10, using the Eq. (17–18).**





**Fig. S2 Dimensionless concentration of (S)-ibuprofen ester $S_{1}(x)$ and (R)-ibuprofen ester $S_{2}(x)$ against the radial distance $x$ by varying the distinct Thiele modulus $\Phi^{2}$ for the conversion degree $X=0,0.99,0.999$ respectively, when the initial bulk concentration is 100 using the Eq. (17–18).**





**Fig. S3 Dimensionless concentration of (S)-ibuprofen ester $S_{1}(x)$ and (R)-ibuprofen ester $S_{2}(x)$ against the radial distance $x$ by varying the distinct racemization constant $\gamma$ for the conversion degree $X=0,0.99,0.999$ when the initial bulk concentration is 100 respectively, using the Eq. (17–18).**
